# The lysosomal disease caused by mutant VPS33A

**DOI:** 10.1093/hmg/ddz077

**Published:** 2019-04-10

**Authors:** Elena V Pavlova, Aleksey Shatunov, Lena Wartosch, Alena I Moskvina, Lena E Nikolaeva, Nicholas A Bright, Karen L Tylee, Heather J Church, Andrea Ballabio, J Paul Luzio, Timothy M Cox

**Affiliations:** 1 Department of Medicine, University of Cambridge, Cambridge, UK; 2 Department of Basic and Clinical Neuroscience, Maurice Wohl Clinical Neuroscience Institute, King’s College London, London, UK; 3 Cambridge Institute for Medical Research and Department of Clinical Biochemistry, Wellcome Trust/MRC Building, University of Cambridge, Cambridge, UK; 4 Paediatric Centre, National Medical Centre of the Republic of Sakha, Yakutsk, Russia; 5 Willink Biochemical Genetics Unit, Genomic Diagnostics Laboratory, Manchester Centre for Genomic Medicine, Central Manchester University Hospitals NHS Foundation Trust, St Mary's Hospital, Manchester, UK; 6 Telethon Institute of Genetics and Medicine, Pozzuoli, Italy

## Abstract

A rare lysosomal disease resembling a mucopolysaccharidosis with unusual systemic features, including renal disease and platelet dysfunction, caused by the defect in a conserved region of the *VPS33A* gene on human chromosome 12q24.31, occurs in Yakuts—a nomadic Turkic ethnic group of Southern Siberia. VPS33A is a core component of the class C core vacuole/endosome tethering (CORVET) and the homotypic fusion and protein sorting (HOPS) complexes, which have essential functions in the endocytic pathway. Here we show that cultured fibroblasts from patients with this disorder have morphological changes: vacuolation with disordered endosomal/lysosomal compartments and—common to sphingolipid diseases—abnormal endocytic trafficking of lactosylceramide. Urine glycosaminoglycan studies revealed a pathological excess of sialylated conjugates as well as dermatan and heparan sulphate. Lipidomic screening showed elevated β-D-galactosylsphingosine with unimpaired activity of cognate lysosomal hydrolases. The 3D crystal structure of human VPS33A predicts that replacement of arginine 498 by tryptophan will de-stabilize VPS33A folding. We observed that the missense mutation reduced the abundance of full-length VPS33A and other components of the HOPS and CORVET complexes. Treatment of HeLa cells stably expressing the mutant VPS33A with a proteasome inhibitor rescued the mutant protein from degradation. We propose that the disease is due to diminished intracellular abundance of intact VPS33A. Exposure of patient-derived fibroblasts to the clinically approved proteasome inhibitor, bortezomib, or inhibition of glucosylceramide synthesis with eliglustat, partially corrected the impaired lactosylceramide trafficking defect and immediately suggest therapeutic avenues to explore in this fatal orphan disease.

## Introduction

A rare and fatal condition resembling a mucopolysaccharidosis (MPS) has been described in infants of *Yakutian* ethnic origin: the principal clinical manifestations include coarse facial features, respiratory obstruction with recurrent pneumonia, skeletal deformities and joint stiffness. In addition to developmental delay, there is splenomegaly, hearing loss, pancytopenia, nephrotic syndrome and renal impairment with a failure to thrive (1). The patients die from sepsis and cardiopulmonary failure in the first 3 years of life. Recently, 13 patients from Yakutia (the Sakha Republic in the Russian Federation) were reported with this ‘MPS plus’ syndrome (OMIM #610034), showing the characteristic clinical features. While increased urinary excretion of glycosaminoglycans (GAGs) compatible with an MPS was detected, no deficiencies of lysosomal enzymes were identified. Whole-exome sequencing in one index family identified a homozygous missense mutation in the *VPS33A* gene, NM_022916.5:c.1492C > T (c.1492C > T);NP_075067.2:p.Arg498Trp (p.R498W) (1). The mutation was verified in other patients by Sanger sequencing. In a separate study, two siblings born into a consanguineous Turkish family had been described with similar features of a lysosomal disease and also harboured the same mutation in the *VPS33A* gene (2).

The finding of this rare variant in Yakut and Turkish patients is consistent with the Turkic ancestry of the present Yakut population (see Discussion). It is estimated that one patient with MPS plus syndrome occurs among the 14–15 000 live births in Yakutia, and the allele frequency of the variant NM_022916.5: c.1492C > T (NP_075067.2:p.Arg498Trp) (p.R498W) was found to be 1 in 110 in a random sample of healthy Yakuts (1).

VPS33A is an orthologue of Vps33p, a protein of the Sec1/Munc18 family, originally discovered in yeast and highly conserved in other eukaryotes (3,4,5). In metazoans, VPS33A is a component of two intracellular heterohexameric membrane-tethering complexes, the class C core vacuole/endosome tethering complex (CORVET), comprising the proteins VPS11, VPS16, VPS18, VPS33A, VPS8 and TGF-BRAP1/TRAP1, and the homotypic fusion and vacuole protein sorting complex (HOPS), comprising the proteins VPS11, VPS16, VPS18, VPS33A, VPS39 and VPS41. While these complexes both participate in the endocytic pathway, CORVET functions in early endosomes (6,7,8) and HOPS is required for tethering and fusion of late endosomes and autophagosomes with lysosomes (9,10,11), as well as the biogenesis of lysosome-related organelles (12). The finding that lysosomes were excessively acidified in fibroblasts from homozygous Yakut VPS33A^R498W^ patients led to the suggestion that VPS33A has an additional function in regulating lysosomal acidification, disruption of which leads to lysosome dysfunction and accumulation of GAGs (1).

Here we report independent studies in five Yakut patients with the MPS-like disease in which we also identified the homozygous mutation NM_022916.5: c.1492C > T (NP_075067.2:p.Arg498Trp) c.1492C > T; p.R498W in the *VPS33A* gene by whole exome sequencing. We have identified further phenotypic characteristics of these patients in which we have discovered abnormal endocytic lactosylceramide trafficking in patient-derived fibroblasts —a common feature of most sphingolipid storage disorders regardless of the primary lysosomal enzyme deficiency (13,14).

Based on the known crystal structure of human VPS33A (15) and immunoblotting of patient-derived fibroblasts and VPS33A^R498W^ expressed in HeLa cells, we propose that the disease results from increased proteasomal degradation of VPS33A^R498W^ leading to reduced abundance and instability of the HOPS and CORVET complexes. Increased degradation of VPS33A was corrected by proteasome inhibitors and the clinically approved proteasome inhibitor, bortezomib, partially rescued lactosylceramide trafficking in patient-derived fibroblasts. Impaired trafficking was also improved by exposure to eliglustat, a selective inhibitor of glucosylceramide biosynthesis.

## Results

### Clinical synopsis

**Figure 1:**
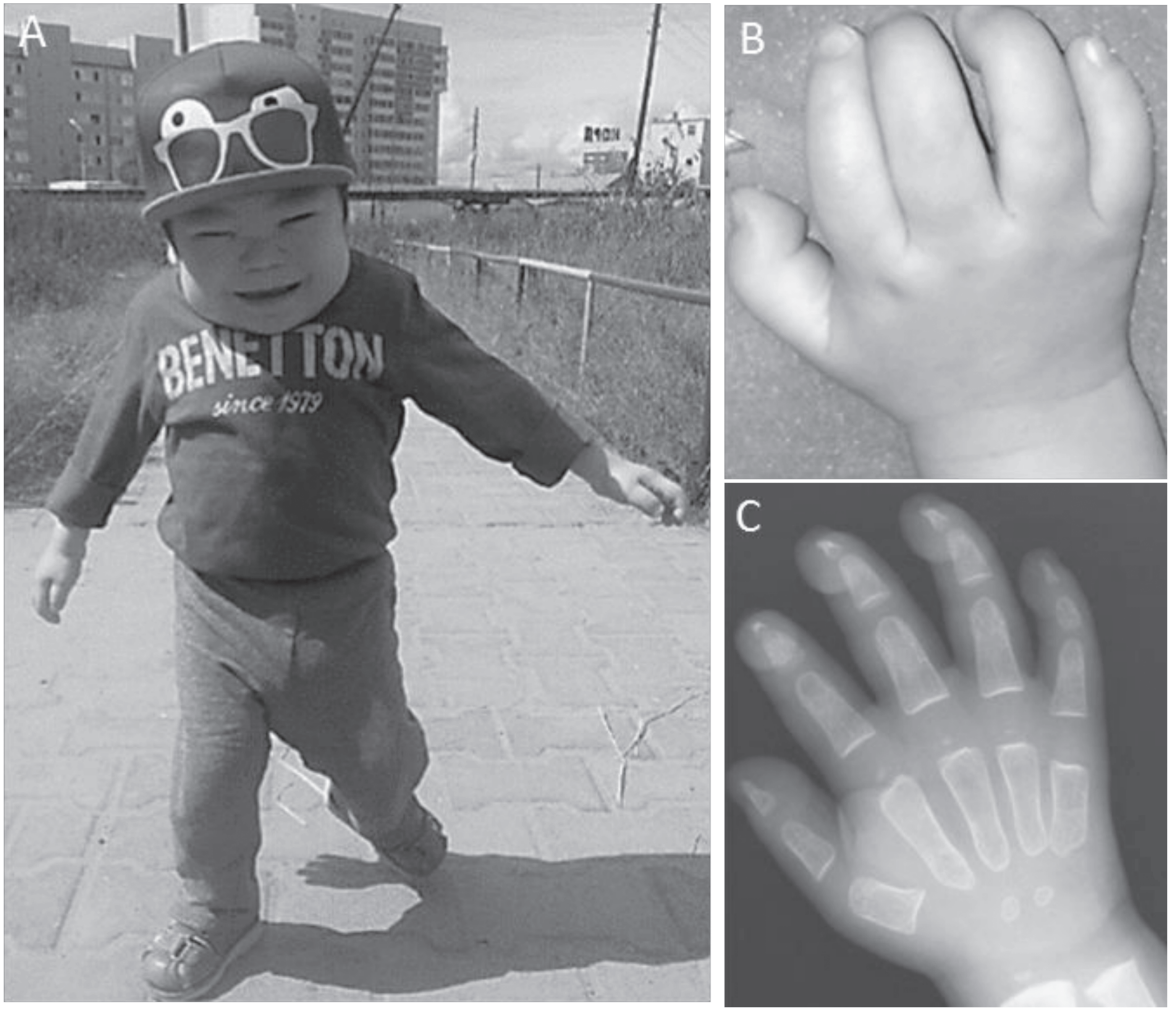
Clinical presentation of the patient P1. (**A**) Coarse facial features, short height, short neck, restricted joints movements and delayed motor development at age 2.9 years. (**B**) Stiff claw-shaped hands. (**C**) Frontal radiograph of the hands at age 16 months: widening of metacarpal bones with proximal pointing.

Five children of Turkic origin in the Republic of Sakha (Yakuts) presented at 4–9 months of age with coarse facial features, short neck and failure to thrive (Fig. 1A). Their illness was characterized by frequent episodes of bronchopneumonia, restricted joint movements and multiple bone deformities. Additional systemic features included nephrotic syndrome with renal impairment (16), retinopathy, thrombocytopenia, leukopenia and anaemia (Table 1, Supplementary Material, Fig. S1). Typical clinical features were claw-shaped hands and broad metacarpal bones, as observed in several mucopolysaccharide diseases (Fig. 1B and C). Severe broncho-pulmonary complications associated with breathlessness occurred in all five patients and one (P5) had suffered a documented apnoeic attack. Other clinical manifestations included irritability and delayed psychomotor and speech development; there were also prominent cerebellar features—hypotonia, poor tendon reflexes and nystagmus.

**Table 1:** Disease manifestations in patients homozygous for the VPS33A p.R498W mutant

Signs/symptoms	Patients	Number affected
Coarse features	P1–P5	5
Recurrent respiratory, intestine infections	P1–P5	5
Frequent conjunctivitis with purulent exudate	P1, P4, P5	3
Splenomegaly	P1–P5	5
Neutropenia	P1, P2, P4, P5	4
Anaemia	P1–P5	5
Thrombocytopenia	P1, P2, P4, P5	4
Coagulation defect	P4, P5	2
Sepsis	P5	1
Patent ductus arteriosus	P4, P5	2
Recurrent bronchopneumonia and chronic obstructive pulmonary disease	P1–P5	5
Motor developmental delay	P1–P5	5
Dysostosis multiplex	P1–P5	5
Joints contraction	P1–P5	5
Nephrotic syndrome	P2, P3, P4, P5	4
Hypoalbuminemia^a^	P1–P5	5
Hyperproteinuria^b^	P1–P5	5
Hypopigmentation of retina	P3	1
High-serum IgM concentration^c^	P1, P3, P4, P5	4
Low-serum IgG concentration^d^	P1, P3, P4, P5	4
Hypotonia	P1–P5	5
Poor tendon reflexes	P1, P2, P3	3
Nystagmus	P3	1

^a^The median of serum albumin concentrations was 26 g/l (range, 19–40 g/l; healthy reference range, 38–54 g/l).

^b^Median of serial urinary protein concentration was 2.57 g/l (range, 0.27–3.3 g/l; in healthy control less than 0.1 g/l).

^c^Range of IgM concentrations was 172–366 mg/dl.

^d^Range of IgG concentrations was 547–840 mg/dl.

Episodes of absolute neutropenia were associated with fever and proven septicaemia associated with otitis media, conjunctivitis or pneumonia caused by gram-positive or gram-negative bacteria. Thrombocytopenia was detected in all four male patients: patients P4 and P5 had severe thrombocytopenia and coagulation defects with episodic intestinal bleeding (Supplementary Material, Fig. S1). In three male patients (P2, P4 and P5), elevated serum IgM concentrations were noted, whereas IgG concentrations were decreased. This hypogammaglobulinaemia may in part account for impaired humoral immunity and susceptibility to recurrent microbial infections. The presence of erythroblasts, myelocytes, metamyelocytes and plasma cells on peripheral blood smears in two patients (P4 and P5) indicates a typical marrow stress response (leukaemoid reaction). Four patients had a full-blown nephrotic syndrome: marked proteinuria causing oedema associated with hypercholesterolaemia, enlarged kidneys and accompanied by ascites as well as pericardial effusions (Table 1). In addition, creatinaemia indicating renal failure, with hypocalcaemia and transient arterial hypertension, was observed.

### Accumulation of GAGs, sialo-glycoconjugates and glycosphingolipids

**Figure 2:**
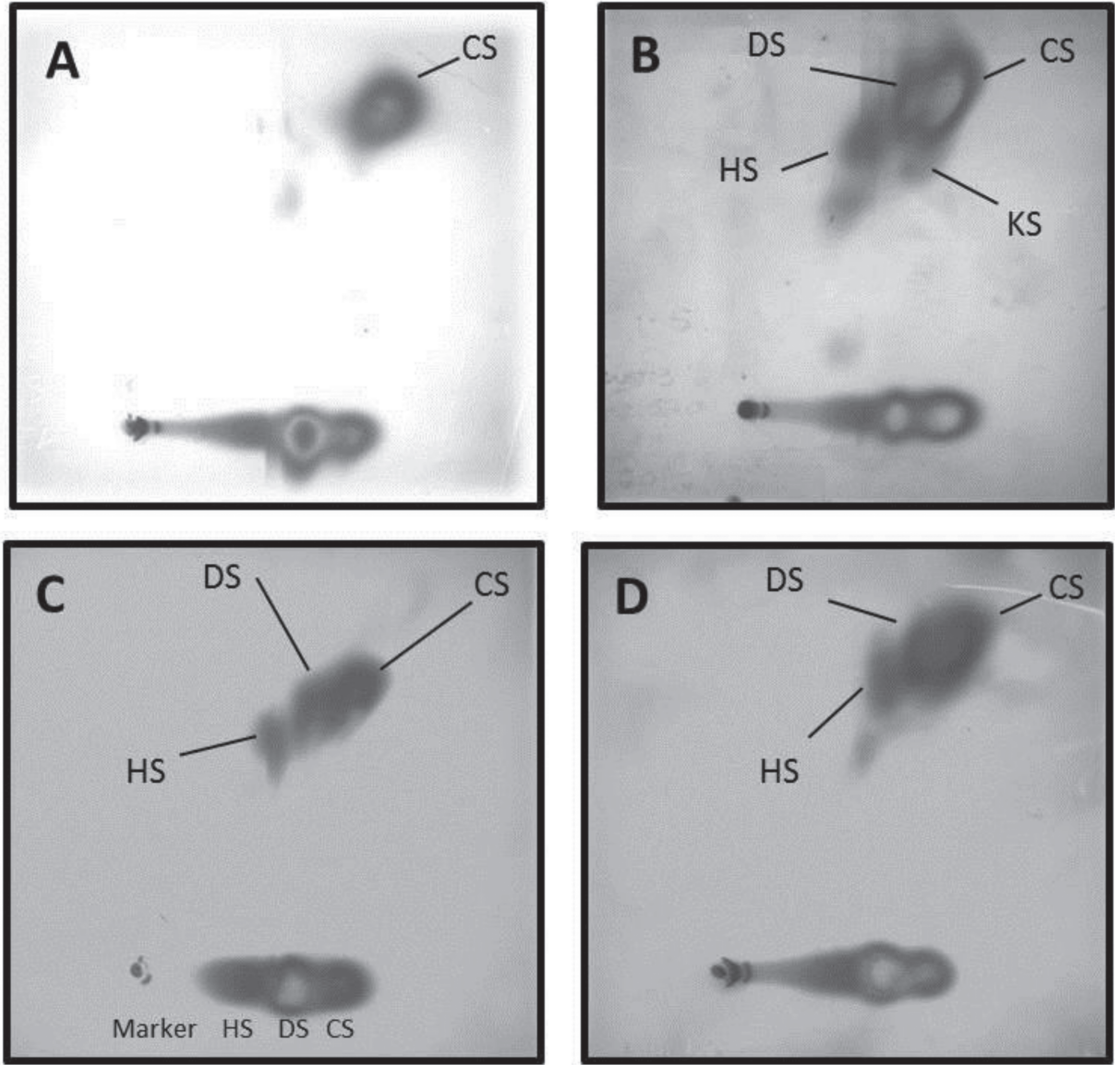
Urinary two-dimensional electrophoresis of GAGs in patients with MPS plus syndrome. (**A**) Healthy individual with normal pattern of GAG presented with chondroitin sulphate (CS). (**B**) Patient P1 urinary GAG pattern showed increased heparan (HS) and dermatan (DS) sulphates with some keratan sulphate (KS). (**C**) Patient P2 urinary pattern with increased heparan (HS) and dermatan (DS) sulphates. (**D**) Patient P3 showed similar pattern of increased heparan (HS) and dermatan (DS) sulphates.

**Figure 3:**
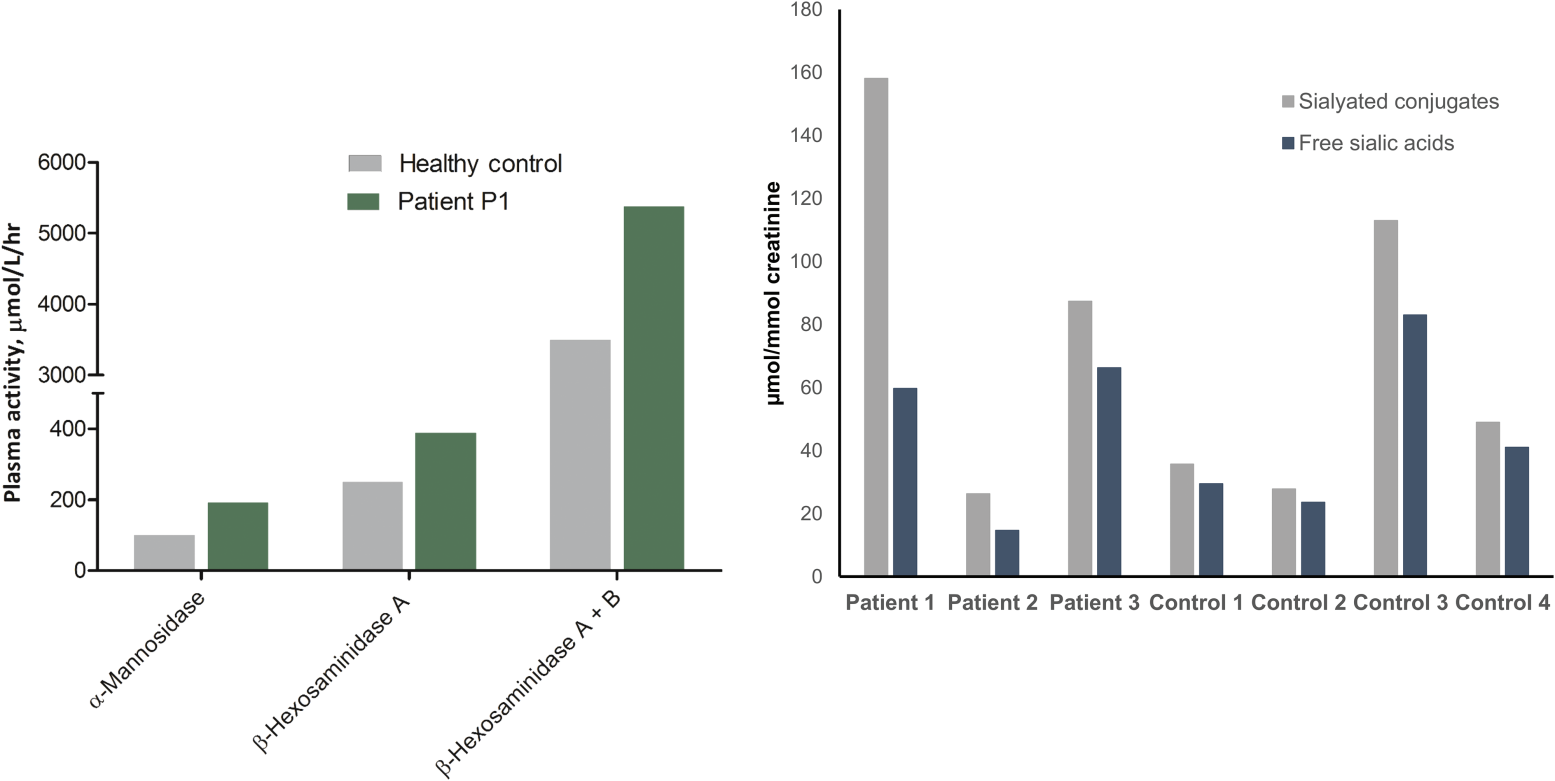
Lysosomal enzymes activities and urinary GAG and sialic acids concentrations in patients with MPS plus syndrome. (**A**) Lysosomal β-hexosaminidase A & B and α-mannosidase activities in plasma of patient P1 and healthy control. (**B**) Urinary excretion of sialylated conjugates and free sialic acids in three patients (P1, P2, P3) and healthy controls presented as sialic acids/creatinine ratio.

The clinical features of the Yakutian patients were strongly suggestive of a lysosomal defect affecting GAG metabolism, but no known specific cause could be identified. Semi-quantitative analysis of urinary GAGs revealed increased heparan sulphate and dermatan sulphates in three patients (P1, P2 and P3; Fig. 2B–D); P1 showed a trace of keratan sulphate. This pattern would typically suggest diagnoses of MPS I, II, MPS VI, MPS VII, mucolipidosis type II/III (ML II/III, I-Cell disease) or possibly multiple sulphatase deficiency. However, the activities of lysosomal acid hydrolases implicated in MPS I, II, VI, VII and several sulphatase enzymes were within the healthy reference ranges in both blood and fibroblasts of patient P1 (Supplementary Material, Table ST1). Plasma β-hexosaminidase A/B, β-hexosaminidase A and α-mannosidase activities in this patient were slightly raised above the reference range but not to the grossly elevated level that would be expected in patients with ML II/III (Fig. 3A, 17).

The activity of α-glucosamine-N-acetyl-transferase was slightly reduced in white cells (0.39 nmol/h/mg; normal range, 0.64–4.2) but normal in fibroblasts (3.2 nmol/h/mg; contemporaneous healthy control, 2.4; affected control, 0.11) isolated from patient P1, making a diagnosis of MPS (Sanfilippo disease) IIIC unlikely. Semi-quantitative sialic acid analysis identified a marked increase of urinary sialoglycoconjugates in patient P1 (Fig. 3B); however, the activity of lysosomal neuraminidase measured in fibroblasts (Supplementary Material, Table ST1) was within the healthy reference range (20 μmol/g/h; normal controls, 12 and 21 μmol/g/h) excluding sialidosis and galactosialidosis, respectively, a lysosomal transporter defect and multienzyme deficiency. Concentrations of free sialic acids in urine were within the age- and sex-matched reference range in all three patients (Fig. 3B).

Mass spectrometry analysis of glucosylceramide, galactosylceramide and sphingolipids in cultured fibroblasts identified an increase of unacylated lyso-glycosphingolipid, β-D-galactosylsphingosine (psychosine), in fibroblasts of patients P1 and P2 (Tables 2 and 3). However, galactosylceramide was lower in patient P1 compared with controls but elevated in patient P2 (Table 2). The activity of lysosomal β-galactosidase, the glycosidase that catalyses removal of the terminal β-galactose from the GM_1_ ganglioside, was in the healthy reference range in patient P1 (Supplementary Material, Table ST1). Concentrations of glucosylceramide isoforms were similar to controls but slightly raised in patient P2 (Table 2). The glucosylceramide/ceramide ratio in both patients was similar to that in control individuals, indicating no disturbance of glucosylceramide metabolism (Table 3). We conclude that these data show abnormal galactosylceramide recycling in patients with this disease and suggest that this may be accompanied by increased de-acylation.

**Table 2:** Glycosphingolipid concentrations in fibroblasts cultured from patients and controls determined by mass spectrometry

Fibroblasts	Glucosylceramide μg/mg Pi	Glucosylsphingosine pmol/mg Pi	Galactosylceramide μg/mg Pi	Galactosylsphingosine pmol/mg Pi
Patient P1	195.2	41.2	0.01	106.9
Patient P2	309.7	81.6	3.25	238.8
Neonatal control	234	26.5	0.27	40.6
Adult control	247.2	64.2	0.78	72.3

**Table 3:** Sphingolipid concentrations in patients with mutated VPS33A and control fibroblasts

Fibroblasts	Sphingosine nmol/mg Pi	Sphingosine-1-P nmol/mg Pi	Total ceramide μg/mg Pi	Glucosylceramide/ceramide ratio
Patient P1	13.0	1.8	59.4	3.3
Patient P2	9.3	0.4	77.3	4
Neonatal control	10.8	1.5	64.5	3.6
Adult control	15.2	2.5	58.7	4.2

### Next-generation exome sequencing and Sanger sequencing

Next-generation targeted sequencing of 89 genes associated with lysosomal, endocytic or autophagic functions did not identify any pathological variants.

Whole exome sequencing of one family trio (two parents and one child P1) and patients P2 and P3 was carried out. Analysis of these five sequenced exomes identified only one homozygous missense mutation, NM_022916.5: c.1492C > T (Supplementary Material, Fig. S2A), in all three patients in a conserved region of the *VPS33A* gene. The transition predicts the replacement of an arginine by tryptophan at position 498 (R498W; NP_075067.2:p.Arg498Trp). Sanger sequencing confirmed segregation of p.R498W as a Mendelian autosomal recessive trait (Supplementary Material, Fig. S2B) since the parents, as well as the unaffected brother of P3, were heterozygous for this mutation. The estimated kinship coefficient predicts that the parents of patient P1 are related to each other with less than a third degree of consanguinity (Supplementary Material, Table ST2).

Sequencing analysis of three patients (P1, P2 and P3) did not identify pathological variants in the GALC gene, encoding galactocerebroside-β-galactosidase, which catalyses degradation of β-galactosylceramide to galactose and ceramide in lysosomes, but a benign variant was found in patient P1 and patient P3 (Supplementary Material, Table ST3) (18). In addition, polymorphisms in the β-galactosidase gene GLB1 were found (Supplementary Material, Table ST3) (19).

### Endosomal/lysosomal phenotype of patient fibroblasts

**Figure 4:**
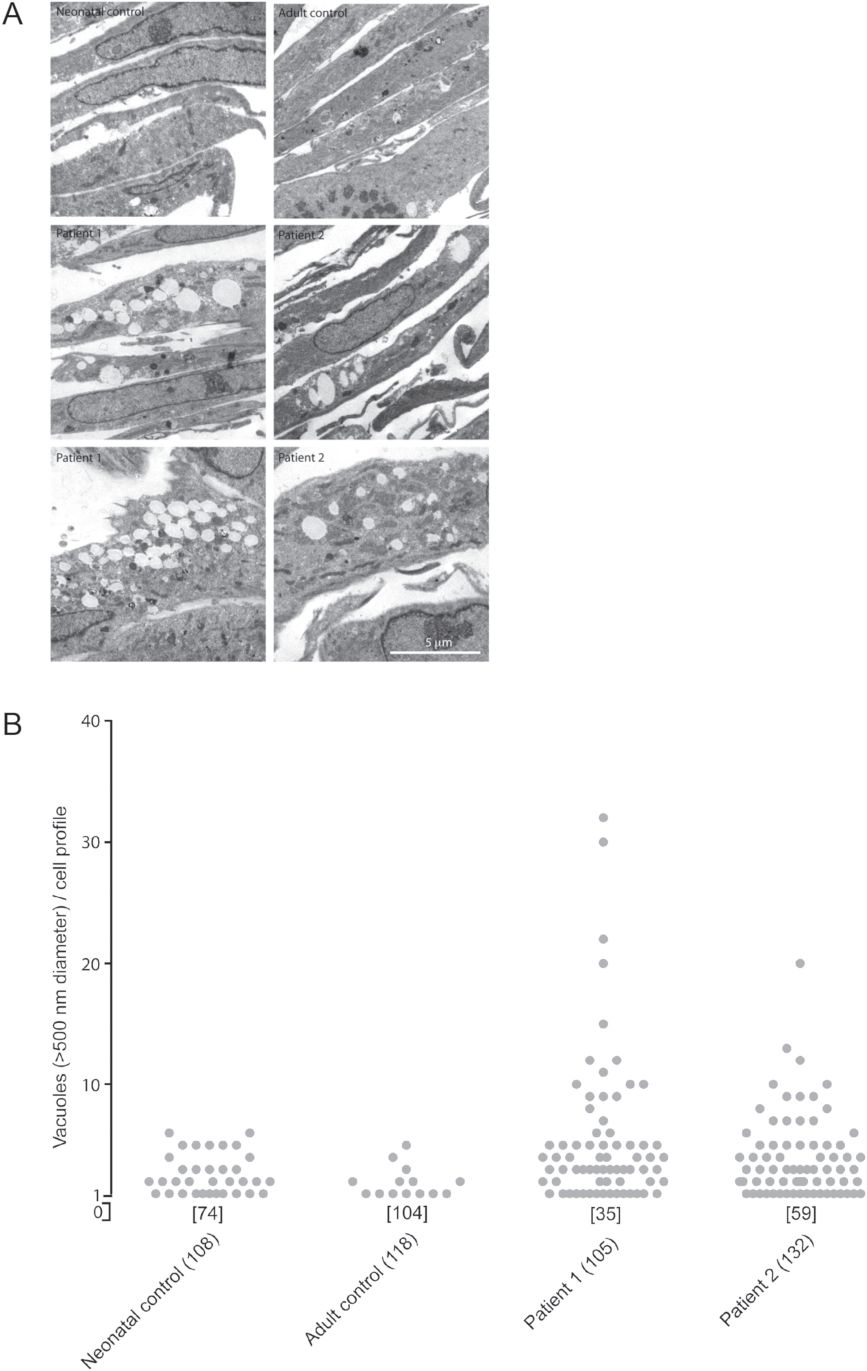
Vacuoles in patient-derived fibroblasts. (**A**) Examples of electron micrographs of thin sections of control and patient-derived (P1, P2) fibroblast pellets. Control fibroblasts had few or no vacuoles. Some patient-derived fibroblasts had many vacuoles. (**B**) Quantification of the number of vacuoles >500 nm diameter per cell profile in thin sections. Number of profiles with no vacuoles indicated in square brackets. Total number of profiles examined in round brackets.

Electron microscopy of skin fibroblasts showed increased vacuolization in those derived from patients compared to wild-type human fibroblasts (Fig. 4A). Quantitative analysis showed that the number of enlarged vacuoles was increased in fibroblasts of both patients analysed (P1 and P2) when compared with controls, consistent with morphological alteration and dysfunction of endosomal/lysosomal compartments (Fig. 4B). Moreover, immunofluorescence confocal microscopy showed that EEA1 fluorescence intensity per cell was increased in the patients’ fibroblasts (Supplementary Material, Fig. S3A–B and E). In contrast, when staining with specific antibodies, the fluorescence intensity of the lysosomal markers LAMP-1 and LAMP-2 was not significantly altered (Supplementary Material, Fig. S4A and B), consistent with previous studies (1). Incubation with the pH indicator, LysoSensor blue dextran DND-167, showed increased fluorescence intensity in patient cells (Supplementary Material, Fig. S4C and D), consistent with the more acidic pH of lysosomes in patient-derived fibroblasts and VPS33A knock-down cells, previously noted (1).

### The effect of the R498W mutation on protein stability

**Figure 5:**
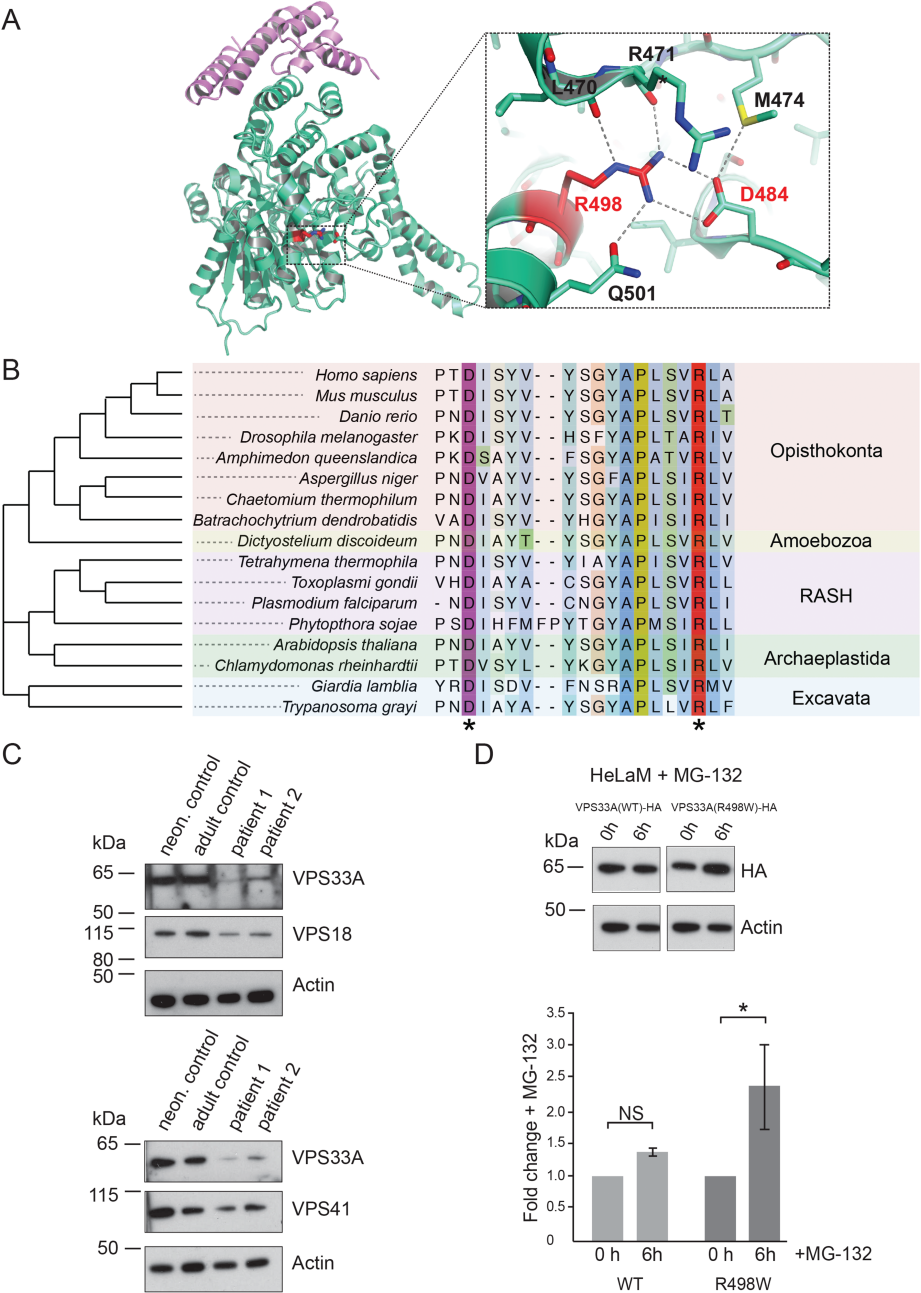
The R498W mutation causes instability of the VPS33A protein. (**A**) Cartoon representation of the crystal structure of human VPS33A (green) in complex with VPS16 (residues 642–736, purple; PDB ID 4xb9; 15). Arginine R498 (highlighted red) forms a salt bridge with aspartate D484 and participates in an extensive network of hydrogen bonds (blow up). (**B**) Phylogenetic tree and alignment of VPS33A show conservation of D484 (purple asterisk) and R498 (red asterisk) among species across all eukaryotic supergroups. RASH: Rhizaria, Alveolata, Stramenopila, Haptophyta. (**C**) Immunoblot analysis showing reduced abundance of VPS33A protein in patient fibroblasts (P1, P2) compared to neonatal (neon) and adult control fibroblasts. In patient fibroblasts, reduced protein levels were also observed for the HOPS/CORVET component VPS18 (upper panel) and the HOPS component VPS41 (lower panel). Actin was used as a loading control. (**D**) HelaM cells stably expressing VPS33A^WT^-HA or VPS33A^R498W^-HA were incubated with 10 μM MG-132 (proteasome inhibitor) for 6 h at 37°C in normal cell culture media (RPMI supplemented with 10% (v/v) FBS, 2 mM glutamine, 100 U/ml penicillin and 100 μg/ml streptomycin) and cell lysates analysed by immunoblotting with anti-HA or anti-actin (loading control) antibodies. Immunoblots from four separate experiments (representative example in upper panel) were scanned and the densities of VPS33A bands quantified and normalized relative to actin. Fold changes in VPS33A concentration are shown relative to no incubation with MG-132 (lower panel). Error bars show SEM. NS, not significant; ^*^, *P* < 0.05.

To predict the consequence of the exchange of arginine for tryptophan at position 498 in VPS33A, we examined the crystal structure of human VPS33A in complex with a fragment of VPS16 (15). In the crystal structure, arginine 498 is located away from the site of interaction with VPS16, but instead forms a salt bridge with an aspartate at position 484. Moreover, it participates in an extensive network of hydrogen bonds (Fig. 5A) possibly further stabilizing the folding in this region. Our analysis showed that the residues D484 and R498 are highly conserved in evolution and are present in representatives of all eukaryotic supergroups (Fig. 5B). Mutation of R498, in domain 2 of VPS33A, to a hydrophobic residue such as tryptophan (W) is predicted to disrupt the salt bridge/hydrogen bond network and weaken the interaction between domains 2 and 3b of VPS33A. This will lead to de-stabilization of the folded structure of the protein and, as a consequence, impaired assembly/stability of the CORVET complex required for early endosome fusion and the HOPS complex required for fusion of endosomes with lysosomes. Consistent with this prediction, immunoblotting of patient fibroblast lysates revealed a reduced abundance of full-length VPS33A (~65 kDa) in the fibroblasts of two patients examined when compared with two different control fibroblast lines (Fig. 5C). This was not a consequence of reduced transcription as relative VPS33A mRNA expression in patients’ fibroblasts was close to that in neonatal control fibroblasts (Supplementary Material, Fig. S5), indicating that the missense mutation R498W does not affect transcription of VPS33A mRNA.

It is noteworthy that we also detected reduced protein abundance of VPS18, another subunit present in the core complex of both CORVET and HOPS, as well as of the HOPS-specific protein VPS41 (Fig. 5C). Expression of GST-tagged VPS33A^R498W^ in *Escherichia coli* failed to yield a sufficient quantity of the mutated protein for biophysical experiments on protein stability (data not shown), despite it being an effective means of generating wild-type protein (data not shown and 15). Although we did not investigate the very low yield of GST-tagged VPS33A^R498W^any further, it is consistent with reduced stability when compared to the wild-type protein. Despite the reduced abundance of VPS33A, VPS18 and VPS41 in the patient-derived fibroblasts, the endocytic uptake and delivery of fluorescent dextran to the cathepsin-active endolysosomal compartment, measured as described previously (10), did not differ statistically in repeated experiments (data not shown). Thus, the reduced concentration of mutated VPS33A^R498W^ in the patients’ fibroblasts was sufficient to support traffic through the endocytic pathway and detectable endosome–lysosome fusion.

To investigate further the effect of the R498W mutation on VPS33A, we stably expressed the HA-tagged R498W mutant of human VPS33A in HeLaM cells and compared it to HA-tagged wild-type VPS33A. The abundance of the mutated protein was less than that of the wild-type protein, when using actin as a reference (Fig. 5D, upper panel). Quantification of data from four separate experiments showed that after exposure to the proteasome inhibitor MG-132 for 6 h, the abundance of VPS33A increased 2.4 ± 0.7-fold in the cells expressing mutant VPS33A (Fig. 5D, lower panel). There was no significant change in the abundance of the protein in wild-type VPS33A-expressing HeLaM cells in the same experiments. These data are consistent with the R498W mutation causing VPS33A protein instability and the mutated protein undergoing post-translational degradation by proteasomes.

### Abnormal lactosylceramide trafficking in the patients’ cells is corrected by the proteasome inhibitor bortezomib

**Figure 6:**
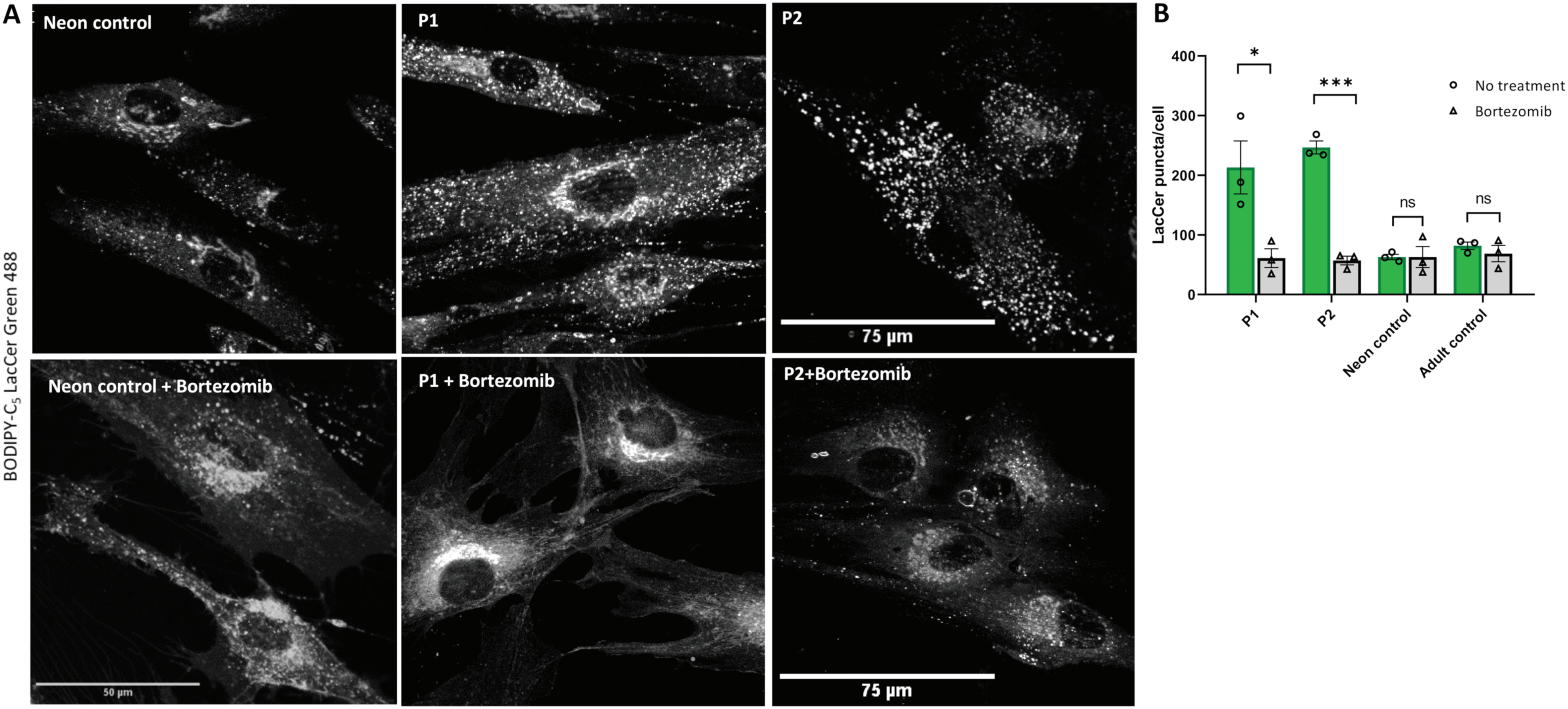
Abnormal BODIPY-LacCer trafficking in patient-derived fibroblasts is corrected by proteasome inhibitor Bortezomib. Patient-derived (P1, P2) or control fibroblasts were grown on 35-mm glass-bottom tissue culture dishes in normal media supplemented with 100 μM of bortezomib (PS-341, Stratech, catalogue number S1013) for 18 h. (**A**) Fibroblasts were labelled with BODIPY-LacCer and endocytosed BODIPY-LacCer was observed on confocal microscope after 3 h uptake. (**B**) Quantification of BODIPY-LacCer puncta in the patient-derived and control cells was performed in *n* ≥ 10 cells per each cell line. Each open circle and triangle represents mean BODIPY-LacCer labelled puncta in untreated and bortezomib treated cells per experiment. The green and grey bars demonstrate mean ± SEM of untreated and bortezomib treated cells from three independent experiments. ^*^, *P* < 0.05; ^***^, *P* < 0.0005; ns, not significant in two-tailed unpaired Student’s *t*-test. Scale bars: 50, 75 μm.

Several unrelated sphingolipid storage diseases have a common sphingolipid trafficking defect associated with lysosomal accumulation of sphingolipids caused by different primary abnormalities (13,14). To explore whether sphingolipid trafficking may be affected by the reduction of VPS33A, we carried out BODIPY C_5_-lactosylceramide (BODIPY-LacCer) pulse labelling and chase experiments in the patients’ and control fibroblasts. In control fibroblasts, after 1-h pulse labelling with BODIPY-LacCer and 2-h chase in normal cell culture medium, BODIPY fluorescence was mainly detected in a perinuclear region consistent with delivery to the Golgi complex as previously reported (13,14,20). In contrast, in the patients’ cells BODIPY fluorescence was observed in more peripheral punctate structures, consistent with accumulation in endocytic organelles, as reported previously in a variety of sphingolipid storage diseases (Fig. 6A; 13,14,20). Moreover, fluorescence in the red spectrum could be detected in the patients’ cells but not in controls (data not shown) consistent with the aggregation-dependent shift from green to red fluorescence emission caused by increasing concentration of lactosylceramide in membranes (13).

**Figure 7:**
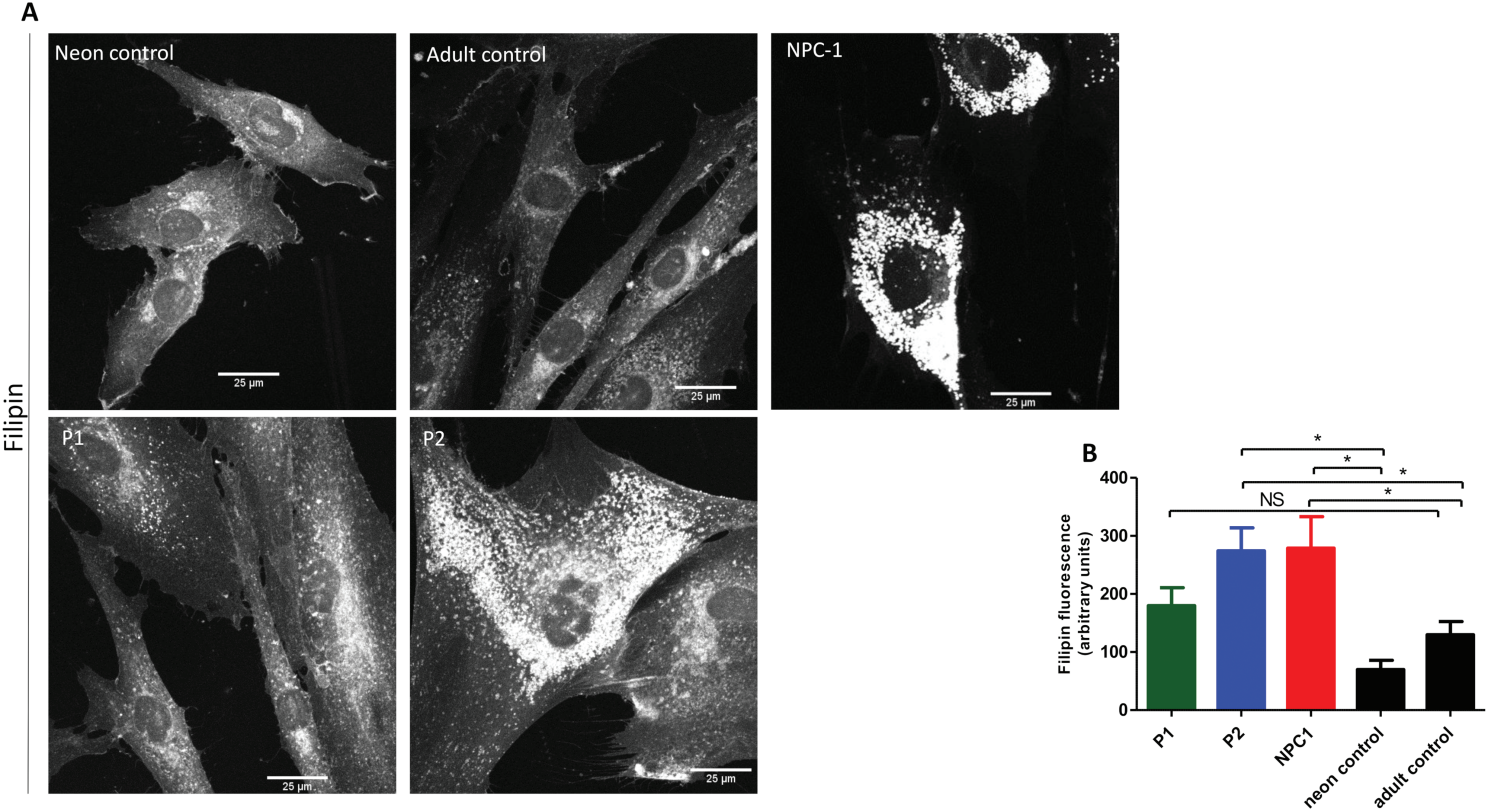
Cholesterol accumulation in patients with MPS plus syndrome. (**A**) Representative confocal images of patients P1, P2, NPC-1 patient and control fibroblasts stained for unesterified cholesterol using filipin. (**B**) Quantification of CTCF was performed in *n* ≥ 10 cells per each cell type. Data presented as mean ± SEM. ^*^, *P* < 0.05 in two-tailed unpaired Student’s *t*-test; NS, not significant.

We further stained the cells with filipin, a fluorescent marker that specifically binds to cholesterol. It is noteworthy that in fibroblasts of patients expressing the VPS33A^R498W^ variant (patients P1 and P2), filipin fluorescence was increased, suggesting increased levels of cholesterol, as found, for example, in fibroblasts from patients with many sphingolipid storage diseases, as well as Niemann–Pick type C fibroblasts (Fig. 7A and B; 14,21).

To test if treatment with a proteasome inhibitor could correct lactosylceramide trafficking, we treated the patients’ cells with the clinically approved drug bortezomib. We found that after exposure to 100 μM of bortezomib for 18 h, BODIPY fluorescence was predominantly found in a perinuclear location and consistent with Golgi localization as seen in normal fibroblasts (Fig. 6A). Quantification of the BODIPY-LacCer fluorescence showed a greatly reduced number of the peripheral puncta in bortezomib-treated patient cells (Fig. 6B). Similar results were obtained after treatment with another proteasome inhibitor, MG-132, although in this case, the number of surviving cells was significantly reduced (data not shown). These observations suggest that the R498W mutation causing instability of VPS33A protein is responsible for disturbed endosomal trafficking of sphingolipids to the Golgi complex and accumulation of sphingolipids in endosomal lysosomal compartments.

### Inhibition of glucosylceramide synthesis by eliglustat improves lactosylceramide trafficking in VPS33A^R498W^ patient-derived cells

Inhibitors of uridine diphosphate (UDP)-glucosylceramide synthase (UDP-glucose: ceramide glucosyltransferase) that catalyses the first step in glycosphingolipid biosynthesis (synthesis of glucosylceramide) are being administered to patients with several sphingolipid lysosomal storage disorders as part of substrate reduction therapy (22,23). Miglustat, N-butyldeoxynojirimycin, has been shown to improve abnormal lipid trafficking in blood lymphocytes in a patient with Niemann–Pick disease type C (NPC) (24); N-butyldeoxygalactonojirimycin has also been demonstrated to reverse disrupted lipid trafficking in a NPC 1 mutant cell line (25). Since we observed abnormal LacCer trafficking in the fibroblasts of patients P1 and P2 (Fig. 6A), we first tested whether miglustat (Zavesca), which is currently available for treatment of NPC, could improve the endocytic trafficking of lactosylceramide in patient-derived fibroblasts. Incubation with 50 μM miglustat showed an improvement in BODIPY-LacCer trafficking in fibroblasts from patient P1 but not in patient P2 (Supplementary Material, Fig. S6A and B).

**Figure 8:**
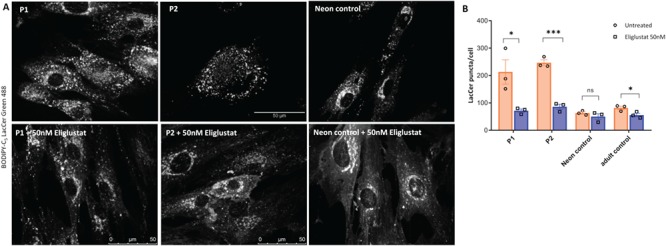
Treatment with eliglustat tartrate, an inhibitor of glucosylceramide synthase, improved Lactosylceramide trafficking in patient-derived fibroblasts. Patient-derived (P1, P2) and control fibroblasts were incubated with 50 nM eliglustat tartrate for 24 h. (**A**) Representative confocal images of patients and control fibroblasts labelled with BODIPY-LacCer as described in the methods. (**B**) Quantification of BODIPY-LacCer puncta in patients and control fibroblasts (*n* ≥ 10 cells per each cell line). Each open circle and square represent mean BODIPY-LacCer labelled puncta in untreated and eliglustat treated cells per experiment. The orange and blue bars indicate mean ± SEM of untreated and eliglustat treated cells from three independent experiments. Scale bars, 75 μm. ^*^, *P* < 0.05; ^***^, *P* < 0.0005; ns, not significant in two-tailed unpaired Student’s *t*-test.

Previously, we and others have shown that treatment with a highly potent and specific inhibitor of UDP-glucosylceramide synthase, eliglustat (Cerdelga) approved for treatment of type 1 Gaucher disease, can reduce glucosylceramide and glucosylsphingosine concentrations (23,26,27,28,29). To test if eliglustat can correct the LacCer trafficking defect, we used 25, 50 and 100 nM concentrations of the drug to expose both patient-derived and control fibroblasts for 24 h before pulse-chase labelling with BODIPY-LacCer. Data from three independent experiments consistently showed an improvement in BODIPY-LacCer trafficking in the cells from the two patients, after incubation with 50 nM eliglustat, as measured by a reduction in BODIPY-LacCer-labelled puncta (Fig. 8A and B). Treatment with 25 nM eliglustat did not improve LacCer trafficking in either patient (Supplementary Material, Fig. S7A and B), whereas incubation with 100 nM showed increased labelling of puncta with BODIPY-LacCer (Supplementary Material, Fig. S7A and C). These results suggest a dose-dependent inhibition of the first step of glycosphingolipid synthesis by eliglustat with correction of lactosylceramide trafficking to the Golgi complex in cells from VPS33A^R498W^ patients.

## Discussion

Our study adds to the molecular understanding of a unique human disease, the ultra-rare MPS plus syndrome, (OMIM #610034), caused by a homozygous missense mutation p.R498W in VPS33A, a core component of the HOPS and CORVET complexes. This mutation was found in the five patients investigated, all of whom were homozygous for the mutation. The patients belong to the principal nomadic Turkic ethnic group in Yakutia, and their clinical features closely resemble those previously described in Yakutian and Turkish patients (1,2). It should be noted that the Yakuts, who are the largest ‘indigenous’ population in a region of Eastern Siberia, have specific anthropological, demographic, linguistic and historical characteristics fully compatible with their historical origin among nomadic Turkic ethnic groups of Central Asia and Southern Siberia (30,31). Genetic studies suggest a bottleneck in the Yakut population: the Yakuts migrated during the 13th–14th centuries CE at the time of the Mongol invasion—ultimately, this population arrived and expanded in North Eastern Siberia. These historical events gave rise to a relatively homogenous Yakut population representing a genetic and geographic isolate with distant relationships to neighbouring native North Siberian ethnic groups (30). The geographic remoteness of the districts, accompanied by extreme arctic weather and seasonal interruptions caused by freezing and thawing of the river Lena and its environs, repeatedly disrupted Yakutian migration. Extreme environmental conditions and isolation are likely to have contributed to inevitable inbreeding and high endogamy accompanied by a high prevalence of autosomal recessive diseases in this population (32). Of note, an increased frequency of several other very rare autosomal recessive diseases has been identified in Yakuts (33,34,35,36).

Patients with MPS plus syndrome have numerous features not shared by the mucopolysaccharidoses and reflect the central role of the HOPS complex in cell physiology: the transient neutropenia associated with susceptibility to bacterial infections, thrombocytopenia and coagulation defects partly resemble a disorder affecting lysosome-related organelles, Chediak–Higashi syndrome. (37,38,39). Peripheral blood lymphocytes with increased granules were reported in Turkish patients (2). These previous observations, together with our data showing vacuolation and increased staining intensity of the early endosomal marker EEA1 in patients’ fibroblasts, provide evidence of enlargement of endosomal/lysosomal organelles in many cell types as well as involvement in secretory granule biogenesis with lysosomal dysfunction in leucocytes and neutrophils. Marked proteinuria and hypoalbuminemia, indicating the nephrotic syndrome, occur in patients with MPS plus syndrome, and proteinuria and vacuolated podocytes with focal segmental glomerulosclerosis have been described in Turkish patients (2). At present, it is not possible to explain how exactly these morphological changes in glomerular epithelial cells cause nephrotic syndrome in patients with MPS plus syndrome, although there are several publications that implicate endocytic pathways in the glomerular filtration barrier function of the kidney (40,41). These studies include an account of conditional inactivation of the murine vacuolar protein sorting 34 (VPS34) gene in renal podocytes: this induces proteinuria, by disrupting the glomerular filtration barrier, and leads to early death (42,43,39,41). VPS34 is a class III phosphoinositide 3-kinase protein, which regulates endocytic trafficking and autophagy and co-localizes with VPS33A (44). Analysis of the VPS34-deficient podocytes showed significant vacuolization, impaired endocytic function and cellular degeneration, thus demonstrating the fundamental role of vesicle trafficking and the endocytic pathway in the pathophysiology of kidney disease (42,43).

We identified a specific urinary GAGs excretion pattern in the patients with mutated VPS33A: abundance of dermatan and heparan sulphates and, in two cases, a unique and unexplained accumulation of sialoglycoconjugates despite unimpaired activities of lysosomal neuraminidase. A similar pattern of urinary GAGs was described in Turkish patients (2), and an elevated total GAGs concentration with greatly increased heparan sulphate were reported by Kondo *et al.* (1) in Yakutian patients’ plasma and fibroblasts as well as in VPS33A siRNA-treated HeLa cells. The pathological urinary GAGs were also present in urine samples from other Turkish patients who had normal lysosomal enzymes activities with a poorly defined MPS but who did not have the c.1492C > T;p.R498W mutation in the *VPS33A* gene (data not shown). Therefore, we are clear that the urinary excretion of GAGs will not constitute a specific diagnostic screening test for MPS plus syndrome. However, patients with suspected MPS who have been identified with the specific urinary GAG and oligosaccharide pattern—increased heparan/dermatan sulphates and sialoglycoconjugates (Figs 2B–D and 3B) but normal activities of related lysosomal enzymes—should be considered for diagnosis of MPS plus syndrome and screened for genetic defects in the *VPS33A* gene.

Here we describe sphingolipid abnormalities in patients homozygous for mutated VPS33A^R498W^. They had an increased concentration of β-D-galactosylsphingosine (psychosine), the de-acylated form of galactosylceramide (Table 2), which is greatly increased in patients with globoid cell leukodystrophy also known as Krabbe disease, or globoid-cell leukodystrophy (45). Psychosine is implicated in loss of oligodendrocytes and defective myelin formation with subsequent and devastating consequences in brain white matter and peripheral nerve roots. Delayed myelination and calcification of basal ganglia, as seen in patients with Krabbe disease and GM_1_ gangliosidosis, had been previously noted in Yakut patients with mutated VPS33A (1). Moreover, a decreased activity of acid β-galactosidase was noted in one Turkish patient with the same mutation in the *VPS33A* gene (2). Sequencing did not find pathological variants in coding parts of the acid β-galactosidase gene (*GALC*), or in the ganglioside β-galactosidase (*GLB1*) gene in three patients (P1, P2, P3) but benign variants were found (Supplementary Material, Table ST3). Activity of the latter was within normal range (Supplementary Material, Table ST1) in patient P1. We consider that the polymorphic variants in *GALC* and *GLB1* genes in Yakutian patients with mutated VPS33A would be unlikely to contribute to the disease, but further studies to understand the relationship, if any, of the VPS33A with lysosomal β-galactosidases would resolve this point definitively.

Unexpectedly, for a disease considered to resemble an MPS, we observed impaired trafficking of sphingolipids after endocytosis. In healthy fibroblasts, BODIPY-LacCer is internalized from the plasma membrane and transported to the Golgi complex via the endosomes but in various sphingolipid storage disorders including, GM_2_, GM_1_ gangliosidosis, prosaposin deficiency, metachromatic leucodystrophy, mucolipidosis type IV, Niemann–Pick types A and C, Krabbe disease and Fabry disease, the BODIPY-LacCer accumulates in endosomes and lysosomes (13,14). Indeed, the trafficking of BODIPY-LacCer was proposed as a screening test for sphingolipid storage diseases (13,14), although the mechanism of defective BODIPY-LacCer trafficking in these otherwise unrelated sphingolipid storage disorders is not understood. In many of these disorders, there was increased filipin staining of the fibroblasts, consistent with increased cholesterol accumulation, but this was not observed in GM_2_ gangliosidosis (Tay–Sachs variant) and was not previously reported in fibroblasts from a VPS33A^R498W^ patient (1). It is important to note that BODIPY-LacCer does not accumulate in fibroblasts of patients with a range of other lysosomal storage diseases in which lipid storage is not characteristic, including MPSII (Hunter disease), Pompe disease, Chediak–Higashi syndrome and alpha-mannosidosis (13). Our observation of defective BODIPY-LacCer trafficking in fibroblasts from VPS33A^R498W^ patients demonstrates that the MPS plus syndrome (OMIM #610034) has the characteristics of a sphingolipid disease as well as an MPS. Importantly, we showed that substrate reduction therapy with 50 nM eliglustat improves LacCer trafficking in VPS33A^R498W^ patients. A dose-response dependent inhibition of the first step of glycosphingolipid synthesis by eliglustat partially corrected LacCer trafficking in VPS33A deficient patient cells. Treatment with 50 μM of miglustat did not revert delayed LacCer trafficking in patient P2, but there was an improvement in patient P1 possibly showing insufficient activity in patient P2 fibroblasts with greater accumulation of cholesterol and glycosphingolipids. Thus, we suggest that a clinical inhibitor of the final step of galactosylceramide synthesis, targeting UDP-galactose ceramide galactosyltransferase might in future be employed for therapeutic substrate reduction of the increased accumulation of galactosylsphingosine in patients with mutated VPS33A.

In fibroblasts from the two VPS33A^R498W^ patient fibroblast lines examined, we observed that while there was no change in the abundance of VPS33A mRNA, the amount of the cognate protein was reduced, when compared to controls. The abundance of VPS18 and VPS41, components of the HOPS complex, was also reduced. Moreover, the reduced abundance of HA-tagged VPS33A^R498W^ compared to wild-type protein, when expressed in HeLaM cells, was rescued by incubation with a proteasome inhibitor. These data are consistent with the reduced stability of the folded R498W mutant protein as predicted from the human VP33A crystal structure (15) and a consequent reduction in the steady-state abundance of the HOPS complex (and probably also the CORVET complex although we did not study this further), which we hypothesize causes the MPS plus syndrome. In mammalian cells, the HOPS complex is required for fusion of lysosomes with endosomes and autophagosomes—as revealed by experiments with HeLaM cells in which endogenous HOPS proteins, including VPS33A, were individually depleted with siRNAs (10,46). It should be noted that at least 80% depletion of the proteins was achieved in those experiments. Our present data showing that endocytic uptake and delivery of fluorescent dextran to the cathepsin-active endolysosomal compartment is not significantly affected in the VPS33A^R498W^ fibroblasts accords with the previous finding that epidermal growth factor receptor endocytosis and degradation is normal in such cells (1). Although impairment of autophagosome–lysosome fusion has also been observed in embryonic fibroblasts from buff (*bf*) mutant mice, which carry the spontaneous point mutation D251E in VPS33A (46), no change in autophagosome–lysosome fusion was reported previously in VPS33A^R498W^ patient-derived fibroblasts (1).

VPS33A and the mammalian HOPS complex are clearly implicated in membrane tethering and fusion in the late endocytic pathway, a molecular mechanism by which they function is inferred from the more extensive studies of the yeast orthologues in vacuole fusion. Thus, in yeast vacuole fusion, Vps33p/HOPS not only acts as a tether but also as a template for the assembly of the soluble N-ethylmaleimide-sensitive factor attachment protein receptors (SNAREs) required for membrane fusion; it also plays a role in SNARE zippering and the terminal stages of fusion (47,48,49,50). From our knowledge of VPS33A/HOPS function in membrane tethering and fusion, the particular challenge is to understand how a single missense mutation R498W and the resultant reduction in abundance of VPS33A and other HOPS proteins induces lysosomal dysfunction—and the accumulation of GAGs in the MPS plus syndrome. We discount the possibility that the mutation prevents VPS33A association with VPS16 as previously suggested (2). We do this not only because of the molecular location of the mutated residue in the crystal structure but also because of experimental evidence that tagged VPS33A^R498W^ co-immunoprecipitates with tagged VPS16 (1). In addition, tagged VPS33A^R498W^ also co-immunoprecipitates with syntaxin 17, suggesting that HOPS interaction with SNAREs is unlikely to be compromised (1).

Preservation of endocytic function and delivery of cargo to endolysosomes of cells with the VPS33A^R498W^ mutation, as well as normal autophagic flux (1), can probably be ascribed to the presence of residual functional HOPS complexes. One previous explanation put forward for the pathological consequences of the VPS33A^R498W^ mutation has been that the decrease in observed mean lysosomal pH, from 4.92 in control fibroblasts to 4.32 in those from homozygous Yakut VPS33A^R498W^ patients, disrupts lysosomal function thus leading to the accumulation of undegraded GAGs and an accompanying MPS phenotype (1). It was proposed that the mutation in VPS33A in effect revealed a previously unknown role for the protein in the direct regulation of lysosomal acidification (1). However, we contend that an alternative explanation for the alteration in pH should be considered, based on the knowledge that the delivery of endocytosed macromolecules to lysosomal hydrolases in mammalian cells requires kiss-and-run and/or complete fusion events between late endosomes (also known as multi-vesicular bodies) and lysosomes to form acidic endolysosomes, in which hydrolytic digestion occurs and from which neutral, re-usable lysosomes are re-formed (51,52,53). The balance of fusion and fission events, which constitute the lysosome fusion and regeneration cycle, regulates the size and acidity of the endolysosomal/lysosomal compartments (53,54). Thus, the reduction in VPS33A/HOPS concentration in cells from VPS33A^R498W^ patients may have a subtle effect on the regulation and/or efficiency of fusion (and a possible knock-on effect on fission) that is not readily observed in short-term experiments on endocytosis. Nevertheless, this can lead to swollen vacuolar compartments and an increase in the proportion of acidic, swollen endolysosomes, causing an overall reduction of mean lysosomal pH and an alteration of the pH profile of endolysosomes/lysosomes in individual cells.

Our hypothesis to explain the effects of the VPS33A^R498W^ mutation takes account of the observation that mean lysosomal pH in a cell or population of cells is the summation of countless different pH values of individual lysosomes, which can be affected by many factors, including intracellular position and state of maturation (53,55). It has the added advantage of providing an explanation for the marked vacuolation in endolysosomal compartments that we observed in patient-derived fibroblasts and was previously noted in patient-derived endothelial cells obtained from conjunctival biopsies (2), since it is well established that altering the balance of fusion and fission events can result in swollen endolysosomes (53,54). One of our most striking observations on the VPS33A^R498W^ patient-derived fibroblasts was the rescue of BODIPY-LacCer trafficking by treatment with the proteasome inhibitor bortezomib. This clearly demonstrates that VPS33A and the HOPS and/or CORVET complexes contribute to the correct delivery of endocytosed glycosphingolipids to the Golgi complex and that the reduction of the VPS33A concentration in the VPS33A^R498W^patients’ cells is sufficient to cause the LacCer trafficking defect without any obvious effect on endocytic delivery to lysosomes.

Genetic defects in known lysosomal sialidases were excluded by sequencing analysis and the concentration of free sialic acids in urine was not increased; this is consistent with normal sialic acid transporter and UDP-N-acetylglucosamine-2-epimerase function. Previous studies have shown that not all lysosomal enzymes have optimum activity at acidic pH. Indeed, lysosomal glycosidases that degrade oligosaccharides occupying the terminal positions of glycoproteins or glycolipids have optimal activity at neutral pH (56) and similarly, a sialic acid-specific O-acetylesterase also has optimal activity in the neutral range (57). It has moreover been shown that dynamic pH changes in endosomal/lysosomal compartments are essential to provide optimal conditions for acidic and neutral lysosomal enzymes and support the sequential denaturation of complex molecules (58,59). We further note that the activity of the lysosomal enzyme β-glucosamine N-acetyl-transferase, which has a wide pH optimum between 5.5 and 7.5 (60,61) and catalyses the breakdown of heparan sulphates, was decreased in leukocytes. We do not know whether impaired trafficking of glycoconjugates occurs in the VPS33A^R498W^ cells, but others have reported that reduced trafficking is associated with an increase in the abundance of glycoconjugates in urine (62).

Taken together with the two recent accounts of this MPS plus syndrome (1,2), our data show that the VPS33A^R498W^ mutation has a profound effect on the morphology and function of endosomal/lysosomal compartments and defective membrane traffic. As well as causing an MPS, it also induces a clinical simulacrum of the discrete family of lysosomal sphingolipid storage diseases due to a novel pathological mechanism that is regulated by the HOPS and/or CORVET complexes. We show that the responsible missense mutation in the VPS33A protein partially destabilizes the HOPS complex, and it is likely to have a similar effect on the CORVET complex. Partial correction of the functional HOPS complex induced by stabilizing VPS33A^R498W^ with a proteasome inhibitor also suggests a therapeutic stratagem based on repurposed clinical agents approved for treating myeloma, such as bortezomib, which target proteasome activity. The salutary effects of eliglustat, a powerful inhibitor of glucosylceramide synthesis on the outer (cytosolic) leaflet of the Golgi that is active in the salvage pathway for re-synthesis of ß-glucosylceramide, offer another independent therapeutic opportunity to explore in this disease. Additional studies will also be needed to fully understand mechanistically how VPS33A^R498W^ leads to vacuolation and swelling of endolysosomal compartments, how it may affect acidification cycling and the consequential diverse clinical syndrome of MPS plus.

## Materials and Methods

### Patients

Five patients, four boys (P1, P2, P4 and P5) and one girl (P3) aged 9–15 months from five (P1–5), not known to be consanguineous, Yakutian families affected by a hitherto unidentified disease were referred internationally for diagnostic advice to the Lysosomal Clinic, Addenbrooke’s Hospital by the Deputy Minister of Republic of Sakha (Yakutia), Ministry of Health and Director of Paediatric National Hospital of Republic Sakha (Yakutia). Four of these patients (P1–4) were recently reported in studies conducted by Kondo *et al.* (1) (respectively, as their patients P12, P13, P9, P1). Retrospective non-identifiable clinical data were analysed according to local and national ethical regulations. Informed consent for genetic studies was obtained from the parents of these patients.

Ethylenediaminetetraacetic acid blood and urine samples were collected for diagnostic investigations, and fibroblasts were established by primary culture of skin biopsies obtained from two patients, P1 and P2. DNA samples were purified from blood or cultured fibroblasts using QiAmp DNA Blood Maxi kit (Qiagen, Netherlands). DNA quality and concentration were determined by Qubit fluorimeter (Life technologies).

### Lysosomal enzyme activities, urine mucopolysaccharide and oligosaccharide analysis

Urinary GAG concentration was determined using a colorimetric protocol as previously described (63). Briefly, GAGs form complex molecules in the presence of the dye 1,9-dimethylmethylene blue (Sigma UK) in acid solution. This produces a colour change from blue to pink, which can be quantified at 520 nm against a standard of known concentration. Extracted GAGs were analysed by two-dimensional electrophoresis on cellulose acetate membranes and then visualized by staining with 0.05% alcian blue stain (Sigma UK) to differentiate sulphated GAG patterns as previously described (64).

Fluorimetric biochemical assays for a range of lysosomal enzyme activities were carried out by clinical diagnostic services at UKAS certified laboratories using commercially available 4-methylumbelliferyl substrates (Glycosynth UK, Carbosynth UK, Moscerdam), including assays for MPS I, MPS II, MPS VI, MPS VII and MPS IIIC (65,66,67).

Oligosaccharide and sialic acid analysis of urine was performed by thin layer chromatography using a previously published protocol (68). Urine oligosaccharides were separated on thin layer silica gel plates using a mobile phase of n-butanol/acetic acid/water (ratio 2:1:1) and then visualized by 0.2% orcinol and heat 100°C. Urine sialic acids were separated on thin layer silica gel plates using an initial mobile phase of n-butanol/acetic acid/water (ratio 2:1:1) followed by propan-1-ol/nitromethane/water (ratio5:4:3) and then visualized by 0.2% orcinol and heat 100°C.

Free sialic acids and sialylated glycoconjugate concentrations were quantified using a previously published colorimetric protocol (69). Free sialic acid (N-acetyl-neuraminic acid) is chemically modified under assay conditions, forming a red pigment in the presence of thiobarbituric acid. This colour change is then extracted in cyclohexanone and quantified by spectroscopy at 549 nm.

### Whole exome sequencing and bioinformatics analysis

Next generation sequencing targeted to 89 lysosomal-endocytic-autophagic disease genes was conducted in the proband P1 and both parents, by means of the LysoPlex_v2 platform (70,71).

Subsequently, whole exome sequencing analysis was carried out in a single nuclear family (two parents and one child P1) and patients P2 and P3 without family members. DNA libraries for exome enrichment analysis were prepared with the Ampliseq Exome kit (Thermo Fisher Scientific) and sequenced on the Ion Proton sequencing system with 200 bp single end reads Hi-Q Chemistry. Alignment of short sequence reads to the published human genome build UCSC hg19 reference sequence was done using the *Torrent Suite 4.4.3* at Genetracer Biotech, Spain.

Variant calling was done using the algorithm implemented in the *samtools 1.3.1* program (72). Generated VCF files were merged in a single dataset by the *bcftoos merge* command and normalized. Duplicates were excluded by the *vt* program (73). Only single nucleotide variants that had a ‘PASS’ tag in the QUAL field of the VCF file with allele frequencies less than 1/1000 and minimum Phred sequencing quality score more than 30 were included in the analysis. To exclude genotyping errors from super-duplicated regions of the genome, the *superdup-filter* option was applied. To filter variants from the quality controlled dataset, we applied the ‘autosomal-recessive full penetrance’ model as implemented in *KGGSeq 1.0* (74). Filtered variants were annotated with the RefGene database compiled by UCSC, the GENCODE gene sets, the NHLBI GO Exome Sequencing Project, the Exome Aggregation Consortium, the Genome Aggregation Database and dbSNP rsID databases.

Variants with the most significant impact were validated by sequencing on an ABI Genetic Analyzer 3730lx (Thermo Fisher Scientific) with Dye Terminator 3.1 reagents (Applied Biosystems). To capture regions of interest, we designed primers (5′-AAAGGCCACAGTCAGGTAGC-3′ and 5′-AAAGAGGTGTATTTAATTCTGACTCC-3′) in the primer3 program using the hg19 build database as a reference sequence (75).

### Kinship coefficient estimation in the nuclear family

We used the KING 2.1.2 program to estimate pairwise kinship coefficients in the index affected family assuming that all members of this family belong to a general population (76). The PLINK 1.9 program (77) was employed to generate binary files suitable for kinship analysis.

### Cell culture

Patients’ dermal fibroblasts were cultured in Minimum Essential Medium Eagle (MEM) (Sigma) supplemented with 10% foetal bovine serum (FBS) (Sigma), 1-mM sodium pyruvate (Sigma), 2 mM Glutamax-1 (Gibco), 100 IU/ml penicillin and 100 μg/ml streptomycin (Invitrogen) at 37°C in a humidified atmosphere of 5% CO_2_. Healthy adult and neonatal fibroblasts were obtained from Sigma (catalogue numbers 106-05A; 106-05N). Fibroblasts from a 1-year old patient with Niemann–Pick type C (GM23162) were from Coriell Institute For Medical Research (New Jersey, USA).

HeLaM cells stably expressing HA-tagged VPS33A^WT^ were generated and cultured as previously described (10) and cells stably expressing HA-tagged VPS33A^R498W^ were generated in the same way, using the pLXIN retroviral system (Clontech).

Cells were incubated with proteasome inhibitors (MG-132 from Sigma or bortezomib from Stratech), or the substrate reduction agents eliglustat tartrate (kindly donated by Professor James Shayman, University of Michigan) or miglustat (Zavesca) donated by patients (incubation times and concentrations as indicated in figure legends).

### Immunoblotting

Lysates of the patients P1 and P2 and control fibroblasts as well as HeLaM cells were used for immunoblot analysis with anti-human VPS11 (WH0055823M1; Sigma-Aldrich), anti-human VPS41 (sc-377 118; Santa Cruz) or antibodies to VPS33A, VPS 18, HA and actin as described previously (10). In addition, lysates from the transfected HeLaM cells were subjected to immunoblot analysis with anti-HA (HA.11, MMS-101R; Covance) and anti-actin (A2066; Sigma-Aldrich) antibodies. In experiments to examine the effects of the proteasome inhibitor MG-132 on HeLaM cells stably expressing VPS33A^WT^-HA or VPS33A^R498W^-HA, immunoblotted VPS33A bands were quantified by densitometry using ImageJ software, normalized to actin bands and fold changes in VPS33A concentration calculated relative to no incubation with MG-132. In each separate experiment, the effect of MG-132 on both VPS33A^WT^-HA and VPS33A^R498W^-HA was measured. A paired student *t-*test was used to calculate *P*-values.

### Quantitative real-time polymerase chain reaction

Total RNA was purified from 1 × 10^6^ patient-derived and neonatal control fibroblasts using the RNeasy Mini kit (Qiagen). The RNA was used for quantitative polymerase chain reaction (PCR) with Taqman gene expression assays for WT VPS33A and Hprt1 (Life Technologies, ThermoFisher Scientific). An RNA-to-CT 1 step kit (Life Technologies ThermoFisher Scientific) was used on an Applied Biosystems 7500 Fast Real Time PCR system accordingly to the manufacturer’s protocol. Three independent TaqMan assays were performed in triplicate and the results were analysed using the comparative ∆∆Ct method (78).

### Transmission electron microscopy

Fibroblast pellets were washed, fixed, stained and analysed by routine transmission electron microscopy as described previously (79), using a FEI Tecnai G2 Spirit BioTWIN transmission electron microscope. The number of vacuoles >500 nm diameter per cell profile in thin (~60 nm) sections was determined.

### Confocal microscopy

Cells were grown on four-well glass slide (Millipore) in normal growth medium for 24 h. Then the cells were fixed, permeabilized using the fixation/permeabilization kit (BD Biosciences catalogue 554 714) and incubated with primary mouse anti-human LAMP1 (BD Biosciences), LAMP2 (Abcam) or EEA-1 (BD Biosciences) monoclonal antibodies and an Alexa Fluor 488 conjugated goat anti-mouse IgG (H + L) antibody. The slides were mounted using ProLong Gold antifade Mountant with DAPI nuclear stain (ThermoFisher Scientific). The slides were observed on a Leica Sp5 confocal microscope, and ≥10 images each containing ≥3 cells were acquired. Images from three independent experiments were analysed using ImageJ software. The fluorescence intensity per cell was quantified in images of maximum intensity Z-projections. The cellular area, the integrated density and the mean grey values were analysed. Measurements of regions without fluorescence were used for background subtraction. The net average fluorescence intensity per pixel, expressed as corrected total cell fluorescence (CTCF), was calculated for each cell. In addition, the cells were stained with LysoSensor Blue DND-167 1 μM or LysoTracker Red DND-99100 nM (Molecular Probes, ThermoFisher Scientific) for 1 h and observed on a Leica SPE confocal microscope.

### Lactosyl ceramide trafficking

Cells were incubated with 5 μM BSA-BODIPY-C_5_lactosylceramide(BODIPY-LacCer) as previously described (20) in serum-free MEM for 1 h at 37°C then washed twice with PBS and incubated with MEM/10% FBS for 2 h at 37°C. Cells were then first washed with PBS and followed by three separate 15 min incubation steps in cold MEM containing 2% fatty acid–free BSA to back-extract plasma membrane lactosyl ceramide. Subsequently, the cells were washed with phenol red-, L-glutamine- and sodium bicarbonate-free MEM containing 1% FCS and 10 mM HEPES, pH 7.4 at room temperature. The cells were imaged on a Leica SPE confocal microscope using solid state lasers at 488 and 532 nm and dichroic filters 488/635 and 405/532 as well as 503–693 nm and 566–726 nm emission filters. For trafficking analysis, 5–10 single live cell images from three separate experiments were acquired and *n* ≥ 10 cells of each cell line were analysed by ImageJ software. For each image, an 8-bit grey scale image was generated using thresholds removing background signals. Pixels clearly corresponding to Golgi fluorescence were selected and removed from the images and puncta containing the remaining pixels were counted.

### Glycosphingolipid analysis

Glycosphingolipids concentrations were quantified in patient and control fibroblast lysates by mass spectrometry. Glycosphingolipids were extracted by the previously described method (80) in the Lipidomics Shared Resource, Hollings Cancer Center, Medical University of South Carolina. Glucosylceramide, galactosylceramide species and sphingolipid concentrations were measured by high-performance liquid chromatography mass spectrometry methodology as previously described (80). Concentrations of glycosphingolipids were normalized to inorganic phosphate (Pi).

## Supplementary Material

HMG-2019-D-00090_Pavlova_Supplementary_Material_revised_clean_ddz077

HMG-2019-D-00090_Pavlova_Supplementary_Material_revised_highlighted_ddz077

## References

[1] Kondo H., Maksimova N., Otomo T., Kato H., Imai A., Asano Y., Kobayashi K., Nojima S., Nakaya A., Hamada Y. et al. (2017) Mutation in VPS33A affects metabolism of glycosaminoglycans: a new type of mucopolysaccharidosis with severe systemic symptoms. *Hum. Mol. Genet.*, 1, 173–183. 10.1093/hmg/ddw377 28013294

[2] Dursun A., Yalnizoglu D., Gerdan O.F., Yucel-Yilmaz D., Sagiroglu M.S., Yuksel B., Gucer S., Sivri S. and Ozgul R.K. (2017) A probable new syndrome with the storage disease phenotype caused by the VPS33A gene mutation. *Clin. Dysmorphol.*, 1, 1–12. 10.1097/MCD.0000000000000149 27547915

[3] Brocker C., Kuhlee A., Gatsogiannis C., Balderhaar H.J., Honscher C., Engelbrecht-Vandre S., Ungermann C. and Raunser S. (2012) Molecular architecture of the multisubunit homotypic fusion and vacuole protein sorting (HOPS) tethering complex. *Proc. Natl. Acad. Sci. U. S. A.*, 109, 1991–1996. 22308417 10.1073/pnas.1117797109 PMC3277535

[4] Rieder S.E. and Emr S.D. (1997) A novel RING finger protein complex essential for a late step in protein transport to the yeast vacuole. *Mol. Biol. Cell.*, 8, 2307–2327. 9362071 10.1091/mbc.8.11.2307 PMC25710

[5] Seals D.F., Eitzen G., Margolis N., Wickner W.T. and Price A. (2000) A Ypt/Rab effector complex containing the Sec1 homolog Vps33p is required for homotypic vacuole fusion. *Proc. Natl. Acad. Sci. U. S. A.*, 97, 9402–9407. 10944212 10.1073/pnas.97.17.9402 PMC16876

[6] Perini E.D., Schaefer R., Stoter M., Kalaidzidis Y. and Zerial M. (2014) Mammalian CORVET is required for fusion and conversion of distinct early endosome sub-populations. *Traffic*, 15, 1366–1389. 25266290 10.1111/tra.12232

[7] Balderhaar H.J. and Ungermann C. (2013) CORVET and HOPS tethering complexes—coordinators of endosome and lysosome fusion. *J. Cell. Sci.*, 126, 1307–1316. 23645161 10.1242/jcs.107805

[8] Lachmann J., Glaubke E., Moore P.S. and Ungermann C. (2014) The Vps39-like TRAP1 is an effector of Rab5 and likely the missing Vps3 subunit of human CORVET. *Cell. Logist.*, 4, e970840. 10.4161/21592780.2014.970840 PMC4325178 25750764

[9] Jiang P., Nishimura T., Sakamaki Y., Itakura E., Hatta T., Natsume T. and Mizushima N. (2014) The HOPS complex mediates autophagosome–lysosome fusion through interaction with syntaxin 17. *Mol. Biol. Cell*, 25, 1327–1337. 24554770 10.1091/mbc.E13-08-0447 PMC3982997

[10] Wartosch L., Günesdogan U., Graham S.C. and Luzio J.P. (2015) Recruitment of VPS33A to HOPS by VPS16 is required for lysosome fusion with endosomes and autophagosomes. *Traffic*, 7, 727–742. 10.1111/tra.12283 PMC4510706 25783203

[11] van der Kant R., Jonker C.T.H., Wijdeven R.H., Bakker J., Janssen L., Klumperman J. and Neefjes J. (2015) Characterization of the mammalian CORVET and HOPS complexes and their modular restructuring for endosome specificity. *J. Biol. Chem.*, 290, 30280–30290. 26463206 10.1074/jbc.M115.688440 PMC4683254

[12] Marks M.S., Heijnen H.F. and Raposo G. (2013) Lysosome-related organelles: unusual compartments become mainstream. *Curr. Opin. Cell Biol.*, 25, 495–505. 23726022 10.1016/j.ceb.2013.04.008 PMC3729921

[13] Chen C.S., Patterson M.C., Wheatley C.L., O'Brien J.F. and Pagano R.E. (1999) Broad screening test for sphingolipid-storage diseases. *Lancet*, 354, 901–905. 10489949 10.1016/S0140-6736(98)10034-X

[14] Puri V., Watanabe R., Dominguez M., Sun X., Wheatley C.L., Marks D.L. and Pagano R.E. (1999) Cholesterol modulates membrane traffic along the endocytic pathway in sphingolipid-storage diseases. *Nat. Cell. Biol.*, 1, 386–388. 10559968 10.1038/14084

[15] Graham S.C., Wartosch L., Gray S.R., Scourfield E.J., Deane J.E., Luzio J.P. and Owen D.J. (2013) Structural basis of Vps33A recruitment to the human HOPS complex by Vps16. *Proc. Natl. Acad. Sci. U. S. A.*, 110, 13345–13350. 23901104 10.1073/pnas.1307074110 PMC3746937

[16] Eddy A.A. and Symons J.M. (2003) Nephrotic syndrome in childhood. *Lancet*, 362, 629–639. 12944064 10.1016/S0140-6736(03)14184-0

[17] Cathey S.S., Leroy J.G., Wood T., Eaves K., Simensen R.J., Kudo M., Stevenson R.E. and Friez M.J. (2010) Phenotype and genotype in mucolipidoses II and III lpha/beta: a study of 61 probands. *J. Med. Genet*., 47, 38–48. 19617216 10.1136/jmg.2009.067736 PMC3712854

[18] Tappino B., Biancheri R., Mort M., Regis S., Corsolini F., Rossi A., Stroppiano M., Lualdi S., Fiumara A., Bembi B. et al. (2010) Identification and characterization of 15 novel GALC gene mutations causing Krabbe disease. *Hum. Mutat*., 31, E1894–E18914. 20886637 10.1002/humu.21367 PMC3052420

[19] Santamaria R., Chabás A., Coll M.J., Miranda C.S., Vilageliu L. and Grinberg D. (2006) Twenty-one novel mutations in the GLB1 gene identified in a large group of GM1-gangliosidosis and Morquio B patients: possible common origin for the prevalent p.R59H mutation among gypsies. *Hum. Mutat.*, 27, 1060. 10.1002/humu.9451 16941474

[20] Pryor P.R., Reimann F., Gribble F.M. and Luzio J.P. (2006) Mucolipin-1 is a lysosomal membrane protein required for intracellular lactosylceramide traffic. *Traffic*, 7, 1388–1398. 16978393 10.1111/j.1600-0854.2006.00475.x PMC7212080

[21] Liscum L. (2000) Niemann–Pick type C mutations cause lipid traffic jam. *Traffic*, 1, 218–225. 11208105 10.1034/j.1600-0854.2000.010304.x

[22] Platt F.M. (2014) Sphingolipid lysosomal storage disorders. *Nature*, 510, 68–75. 24899306 10.1038/nature13476

[23] McEachern K.A., Fung J., Komarnitsky S., Siegel C.S., Chuang W.L., Hutto E., Shayman J.A., Grabowski G.A., Aerts J.M., Cheng S.H. et al. (2007) A specific and potent inhibitor of glucosylceramide synthase for substrate inhibition therapy of Gaucher disease. *Mol. Genet. Metab.*, 91, 259–267. 17509920 10.1016/j.ymgme.2007.04.001

[24] Lachmann R.H., teVruchte D., Lloyd-Evans E., Reinkensmeier G., Sillence D.J., Fernandez-Guillen L., Dwek R.A., Butters T.D., Cox T.M. and Platt F.M. (2004) Treatment with miglustat reverses the lipid-trafficking defect in Niemann–Pick disease type C. *Neurobiol. Dis.*, 16, 654–658. 15262277 10.1016/j.nbd.2004.05.002

[25] teVruchte D., Lloyd-Evans E., Veldman R.J., Neville D.C., Dwek R.A., Platt F.M., van Blitterswijk W.J. and Sillence D.J. (2004) Accumulation of glycosphingolipids in Niemann–Pick C disease disrupts endosomal transport. *J. Biol. Chem.*, 279, 26167–26175. 15078881 10.1074/jbc.M311591200

[26] Shayman J.A. (2015) Developing novel chemical entities for the treatment of lysosomal storage disorders: an academic perspective. *Am. J. Physiol. Renal. Physiol*., 309, F996–F999. 26447223 10.1152/ajprenal.00393.2015

[27] Cox T.M. (2010) Eliglustat tartrate, an orally active glucocerebroside synthase inhibitor for the potential treatment of Gaucher disease and other lysosomal storage diseases. *Curr. Opin. Investig. Drugs.*, 11, 1169–1181. 20872320

[28] Cox T.M., Drelichman G., Cravo R., Balwani M., Burrow T.A., Martins A.M., Lukina E., Rosenbloom B., Ross L., Angell J. et al. (2015) Eliglustat compared with imiglucerase in patients with Gaucher's disease type 1 stabilised on enzyme replacement therapy: a phase 3, randomised, open-label, non-inferiority trial. *Lancet*, 385, 2355–1262. 25819691 10.1016/S0140-6736(14)61841-9

[29] Pavlova E.V., Archer J., Wang S., Dekker N., Aerts J.M., Karlsson S. and Cox T.M. (2015) Inhibition of UDP-glucosylceramide synthase in mice prevents Gaucher disease-associated B-cell malignancy. *J. Pathol.*, 235, 113–124. 25256118 10.1002/path.4452

[30] Pakendorf B., Novgorodov I.N., Osakovskij V.L., Danilova A.P., Protod′jakonov A.P. and Stoneking M. (2006) Investigating the effects of prehistoric migrations in Siberia: genetic variation and the origins of Yakuts. *Hum. Genet.*, 120, 334–353. 16845541 10.1007/s00439-006-0213-2

[31] Fedorova S.A. and Khusnutdinova E.K. (2010) Gene pools of peoples from the Republic Sakha (Yakutia): structure, origin, genetic relationships. *Russ. J. Genet.*, 46, 1244–1246. 21061627

[32] Tarskaya L.A., El’chinova G.I. and Vinokurov V.N. (2003) Analysis of marital migrations in two regions in the Sakha Republic (Iakutia). *Russ. J. Genet.*, 39, 1698–1701. 14964839

[33] Barashkov N.A., Dzhemileva L.U., Fedorova S.A., Teryutin F.M., Posukh O.L., Fedotova E.E., Lobov S.L. and Khusnutdinova E.K. (2011) Autosomal recessive deafness 1A (DFNB1A) in Yakut population isolate in Eastern Siberia: extensive accumulation of the splice site mutation IVS1+1G>A in GJB2 gene as a result of founder effect. *J. Hum. Genet.*, 56, 631–639. 21776002 10.1038/jhg.2011.72

[34] Maksimova N., Hara K., Miyashia A., Nikolaeva I., Shiga A., Nogovicina A., Sukhomyasova A., Argunov V., Shvedova A., Ikeuchi T. et al. (2007) Clinical, molecular and histopathological features of short stature syndrome with novel CUL7 mutation in Yakuts: new population isolate in Asia. *J. Med. Genet.*, 44, 772–778. 17675530 10.1136/jmg.2007.051979 PMC2652813

[35] Maksimova N.R., Nikolaeva I.A., Korotkov M.N., Ikeuchi T., Onodera O., Nishizava M., Stepanova S.K., Kurtanov K.A., Sukhomiasova A.L., Nogovitsyna A.N. et al. (2008) The clinical-genealogic and molecular-genetic characteristics of oculopharyngeal muscular dystrophy in the Republic of Sakha (Yakutia). *Zh. Nevrol. Psikhiatr. Im. S. S. Korsakova*, 108, 52–60. 18577936

[36] Galeeva N.M., Nazarenko L.P., Nazarenko S.A., Tverskaya S.M. and Polyakov A.V. (2005) Molecular-genetic cause of recessive congenital methemoglobinemia type 1 in Yakutia. *Med. Genet*., 9, 15–21.

[37] Spicer S.S., Sato A., Vincent R., Eguchi M. and Poon K.C. (1981) Lysosome enlargement in the Chediak–Higashi syndrome. *Fed. Proc.*, 40, 1451–1455. 7215561

[38] Stinchcombe J.C., Page L.J. and Griffiths G.M. (2000) Secretory lysosome biogenesis in cytotoxic T lymphocytes from normal and Chediak Higashi syndrome patients. *Traffic*, 1, 435–444. 11208129 10.1034/j.1600-0854.2000.010508.x

[39] Luzio J.P., Hackmann Y., Dieckmann N.M. and Griffiths G.M. (2014) The biogenesis of lysosomes and lysosome-related organelles. *Cold Spring Harb. Perspect. Biol.*, 6, a016840. 10.1101/cshperspect.a016840 PMC4142962 25183830

[40] Carson J.M., Okamura K., Wakashin H., McFann K., Dobrinskikh E., Kopp J.B. and Blaine J. (2014) Podocytes degrade endocytosed albumin primarily in lysosomes. *PLoS One*, 9, e99771. 10.1371/journal.pone.0099771 PMC4055698 24924335

[41] Soda K. and Ishibe S. (2013) The function of endocytosis in podocytes. *Curr. Opin. Nephrol. Hypertens.*, 22, 432–438. 23703394 10.1097/MNH.0b013e3283624820 PMC4143890

[42] Chen J., Chen M.X., Fogo A.B., Harris R.C. and Chen J.K. (2013) mVps34 deletion in podocytes causes glomerulosclerosis by disrupting intracellular vesicle trafficking. *J. Am. Soc. Nephrol.*, 24, 198–207. 23291473 10.1681/ASN.2012010101 PMC3559479

[43] Bechtel W., Helmstädter M., Balica J., Hartleben B., Kiefer B., Hrnjic F., Schell C., Kretz O., Liu S., Geist F. et al. (2013) Vps34 deficiency reveals the importance of endocytosis for podocyte homeostasis. *J. Am. Soc. Nephrol.*, 24, 727–743. 23492732 10.1681/ASN.2012070700 PMC3636793

[44] Hunter M.R., Hesketh G.G., Benedyk T.H., Gingras A.C. and Graham S.C. (2018) Proteomic and biochemical comparison of the cellular interaction partners of human VPS33A and VPS33B. *J. Mol. Biol*., 430, 2153–2163. 29778605 10.1016/j.jmb.2018.05.019 PMC6005816

[45] Svennerholm L., Vanier M.T. and Månsson J.E. (1980) Krabbe disease: a galactosylsphingosine (psychosine) lipidosis. *J. Lipid. Res.*, 21, 53–64. 7354254

[46] Zhen Y. and Li W. (2015) Impairment of autophagosome–lysosome fusion in the buff mutant mice with the VPS33A(D251E) mutation. *Autophagy*, 11, 1608–1622. 26259518 10.1080/15548627.2015.1072669 PMC4590608

[47] Baker R.W., Jeffrey P.D., Zick M., Phillips B.P., Wickner W.T. and Hughson F.M. (2015) A direct role for the Sec1/Munc18-family protein Vps33 as a template for SNARE assembly. *Science*, 349, 1111–1114. 26339030 10.1126/science.aac7906 PMC4727825

[48] Orr A., Song H., Rusin S.F., Kettenbach A.N. and Wickner W. (2017) HOPS catalyzes the interdependent assembly of each vacuolar SNARE into a SNARE complex. *Mol. Biol. Cell*., 28, 975–983. 28148647 10.1091/mbc.E16-10-0743 PMC5385945

[49] Schwartz M.L., Nickerson D.P., Lobingier B.T., Plemel R.L., Duan M., Angers C.G., Zick M. and Merz A.J. (2017) Sec17 (α-SNAP) and an SM-tethering complex regulate the outcome of SNARE zippering in vitro and in vivo. *Elife*, 6, e27396. 10.7554/eLife.27396 PMC5643095 28925353

[50] D'Agostino M., Risselada H.J., Lürick A., Ungermann C. and Mayer A. (2017) A tethering complex drives the terminal stage of SNARE-dependent membrane fusion. *Nature*, 551, 634–638. 29088698 10.1038/nature24469

[51] Luzio J.P., Pryor P.R. and Bright N.A. (2017) Lysosomes: fusion and function. *Nat. Rev. Mol. Cell Biol*., 8, 622–632. 10.1038/nrm2217 17637737

[52] Huotari J. and Helenius A. (2011) Endosome maturation. *EMBO J.*, 30, 3481–3500. 21878991 10.1038/emboj.2011.286 PMC3181477

[53] Bright N.A., Davis L.J. and Luzio J.P. (2016) Endolysosomes are the principal intracellular sites of acid hydrolase activity. *Curr. Biol.*, 26, 2233–2245. 27498570 10.1016/j.cub.2016.06.046 PMC5026700

[54] Bissig C., Hurbain I., Raposo G. and van Niel G. (2017) PIKfyve activity regulates reformation of terminal storage lysosomes from endolysosomes. *Traffic*, 18, 747–757. 28857423 10.1111/tra.12525

[55] Johnson D.E., Ostrowski P., Jaumouillé V. and Grinstein S. (2016) The position of lysosomes within the cell determines their luminal pH. *J. Cell Biol.*, 212, 677–692. 26975849 10.1083/jcb.201507112 PMC4792074

[56] Butor C., Griffiths G., Aronson N.N. Jr. and Varki A. (1995) Co-localization of hydrolytic enzymes with widely disparate pH optima: implications for the regulation of lysosomal pH. *J. Cell Sci*., 108, 2213–2219. 7673341 10.1242/jcs.108.6.2213

[57] Butor C., Higa H.H. and Varki A. (1993) Structural, immunological, and biosynthetic studies of a sialic acid-specific O-acetylesterase from rat liver. *J. Biol. Chem*., 268, 10207–10213. 8486688

[58] Aronson N.N.J. and Kuranda M.J. (1989) Lysosomal degradation of Asn-linked glycoproteins. *FASEB J.*, 3, 2615–2622. 2531691 10.1096/fasebj.3.14.2531691

[59] Higa H.H., Manzi A. and Varki A. (1989) O-acetylation and de-O-acetylation of sialic acids. Purification, characterization, and properties of a glycosylated rat liver esterase specific for 9-O-acetylated sialic acids. *J. Biol. Chem.*, 264, 19435–19442. 2808434

[60] Pallini R., Leder I.G. and di Natale P. (1984) Sanfilippo type C diagnosis: assay of acetyl-CoA: alpha-glucosaminide N-acetyltransferase using [14C]glucosamine as substrate and leukocytes as enzyme source. *Pediatr. Res.*, 18, 543–545. 6429622 10.1203/00006450-198406000-00013

[61] Choi Y., Tuzikov A.B., Ovchinnikova T.V., Bovin N.V. and Pshezhetsky A.V. (2016) Novel direct assay for acetyl-CoA:α-glucosaminide *N*-acetyltransferase using BODIPY-glucosamine as a substrate. *JIMD Reports.*, 28, 11–18. 10.1007/8904_2015_501 PMC5059212 26493749

[62] Waters P.J., Lewry E. and Pennock C.A. (1992) Measurement of sialic acid in serum and urine: clinical applications and limitations. *Ann. Clin. Biochem.*, 29, 625–637. 1489159 10.1177/000456329202900603

[63] De Jong J.G., Wevers R.A. and Liebrand-van Sambeek R. (1992) Measuring urinary glycosaminoglycans in the presence of protein: an improved screening procedure for mucopolysaccharidoses based on dimethylmethylene blue. *Clin. Chem.*, 38, 803–807. 1597005

[64] Wessler E. (1968) Analytical and preparative separation of acidic glycosaminoglycans by electrophoresis in barium acetate. *Anal. Biochem.*, 26, 39–44. 10.1016/0003-2697(68)90205-4 5716194

[65] Stirling J.L., Robinson D., Fensom A.H., Benson P.F. and Baker J.E. (1978) Fluorimetric assay for prenatal detection of Hurler and Scheie homozygotes or heterozygotes. *Lancet*, 1, 147. 10.1016/s0140-6736(78)90441-5 87574

[66] Voznyi Y.V., Keulemans J.L.M. and van Diggelen O.P. (2001) A fluorogenic assay for the diagnosis of Hunter disease (MPS II). *J. Inher. Metab. Dis.*, 24, 675–680. 11768586 10.1023/a:1012763026526

[67] Fluharty A.L., Stevens R.L., Sanders D.L. and Kihara H. (1974) Arylsulphatase B deficiency in Maroteaux–Lamy Syndrome cultured fibroblasts. *Biochem. Biophys. Res. Commun.*, 59, 455–461. 4277366 10.1016/s0006-291x(74)80001-x

[68] Sewell A.C. (1979) An improved thin-layer chromatographic method for urinary oligosaccharide screening. *Clinica Chimica Acta*, 92, 411–414. 10.1016/0009-8981(79)90221-3 285767

[69] Roboz J., Suttajit M. and Bekesi J.G. (1981) Elimination of 2-deoxyribose interference in the thiobarbituric acid determination of N-acetylneuraminic acid in tumor cells by pH-dependent extraction with cyclohexanone. *Anal. Biochem.*, 110, 380–388. 7235225 10.1016/0003-2697(81)90207-4

[70] Di Fruscio G., Schulz A., De Cegli R., Savarese M., Mutarelli M., Parenti G., Banfi S., Braulke T., Nigro V. and Ballabio A. (2015) Lysoplex: an efficient toolkit to detect DNA sequence variations in the autophagy-lysosomal pathway. *Autophagy*, 11, 928–938. 26075876 10.1080/15548627.2015.1043077 PMC4502703

[71] Di Fruscio G., Banfi S., Nigro V. and Ballabio A. (2017) Next-generation sequencing approaches to define the role of the autophagy lysosomal pathway in human disease: the example of LysoPlex. *Methods Mol. Biol.*, 1594, 227–241. 28456987 10.1007/978-1-4939-6934-0_15

[72] Li H., Handsaker B., Wysoker A., Fennell T., Ruan J., Homer N., Marth G., Abecasis G., Durbin R. and 1000 Genome Project Data Processing Subgroup (2009) The Sequence alignment/map (SAM) format and SAMtools. *Bioinformatics*, 25, 2078–2079. 19505943 10.1093/bioinformatics/btp352 PMC2723002

[73] Tan A., Abecasis G.R. and Kang H.M. (2015) Unified representation of genetic variants. *Bioinformatics*, 31, 2202–2204. 25701572 10.1093/bioinformatics/btv112 PMC4481842

[74] Li M., Li J., Li M.J., Pan Z., Hsu J.S., Liu D.J., Zhan X., Wang J., Song Y. and Sham P.C. (2017) Robust and rapid algorithms facilitate large-scale whole genome sequencing downstream analysis in an integrative framework. *Nucleic Acids Res.*, 45, e75. 28115622 10.1093/nar/gkx019 PMC5435951

[75] Untergrasser A., Cutcutache I., Koressaar T., Ye J., Faircloth B.C., Remm M. and Rozen S.G. (2012) Primer3—new capabilities and interfaces. *Nucleic Acids Res*., 40, e115. 22730293 10.1093/nar/gks596 PMC3424584

[76] Chang C.C., Chow C.C., Tellier L.C.A.M., Vattikuti S., Purcell S.M. and Lee J.J. (2015) Second-generation PLINK: rising to the challenge of larger and richer datasets. *GigaScience*, 4, 1–16. 25722852 10.1186/s13742-015-0047-8 PMC4342193

[77] Manichaikul A., Mychaleckyj J.C., Rich S.S., Daly K., Sale M. and Chen W.M. (2010) Robust relationship inference in genome-wide association studies. *Bioinformatics*, 26, 2867–2873. 20926424 10.1093/bioinformatics/btq559 PMC3025716

[78] Livak K.J. and Schmittgen T.D. (2001) Analysis of relative gene expression data using real-time quantitative PCR and the 2(-Delta Delta C(T)) Method. *Methods*, 25, 402–408. 11846609 10.1006/meth.2001.1262

[79] Bright N.A., Reaves B.J., Mullock B.M. and Luzio J.P. (1997) Dense core lysosomes can fuse with late endosomes and are re-formed from the resultant hybrid organelles. *J. Cell Sci*., 110, 2027–2040. 9378754 10.1242/jcs.110.17.2027

[80] Bielawski J., Pierce J.S., Snider J., Rembiesa B., Szulc Z.M. and Bielawska A. (2009) Comprehensive quantitative analysis of bioactive sphingolipids by high-performance liquid chromatography–tandem mass spectrometry In Armstrong D. (ed), *Lipidomics. Methods in Molecular Biology (Methods and Protocols)*. Humana Press, Totowa, NJ, Vol. 579, pp. 443–467. 10.1007/978-1-60761-322-0_22 19763489

