# Supplementary material for: The lysosomal disease caused by mutant VPS33A
Source: Hum Mol Genet. 2019 Apr 10;28(15):2514–30. doi: 10.1093/hmg/ddz077 (PMC6644154; doi:10.1093/hmg/ddz077)
Supplement: HMG-2019-D-00090_Pavlova_Supplementary_Material_revised_highlighted_ddz077 [file hmg-2019-d-00090_pavlova_supplementary_material_revised_highlighted_ddz077.docx]

| **Supplementary Material**  **Table ST1. Lysosomal enzymes activities in patient P1.** | |  |  |  |  |
| --- | --- | --- | --- | --- | --- |
| **Willink Biochemical Genetics Unit** | |  |  |  |  |
| **Tissue** | **Enzyme** | **Activity** | **Control** | **Reference Range** | **Result comment** |
| Leucocyte | Iduronidase | 19 µmol/g/hr |  | 10-50 umol/g/hr | Normal |
| Leucocyte | Glucuronidase | 170 µmol/g/hr |  | 100-800 umol/g/hr | Normal |
| Plasma | IDS | 564 nmol/ml/4 hr |  | 494-113 nmol/ml/4 hr | Normal |
| Plasma | α-Mannosidase | 192 µmol/l/hr | 100 | 20-100 umol/l/hr | Raised |
| Plasma | Hexosaminidase A + B | 389 µmol/l/hr | 250 | 50-250 umol/l/hr | Raised |
| Plasma | Hexosaminidase A | 5380 µmol/l/hr | 3500 | 600-3500 umol/l/hr | Raised |
| Fibroblasts | Arylsulphatase B | 90 µmol/g/hr | Aff = 4, Norm = 32 and 91 |  | Normal |
| Fibroblasts | α-Mannosidase | 32 µmol/g/hr | Norm = 19 and 24 |  | Normal |
| Fibroblasts | β-Galactosidase | 679 µmol/g/hr | Norm = 357 and 717 |  | Normal |
| Fibroblasts | β- Glucosidase | 163 µmol/g/hr | Norm = 144 1nd 215 |  | Normal |
| Fibroblasts | Neuraminidase | 20 µmol/g/hr | Aff = 0.28, Norm = 12 and 21 |  | Normal |
| Fibroblasts | Sulphamidase | 85.9 nmol/mg/17hrs | Norm=82.5; affected=1.2 |  | Normal |
| Fibroblasts | Alpha-N-glucosaminidase | 14.9 nmol/mg/hr | norm=7.7' affected=0.2 |  | Normal |
| Fibroblasts | α-Glucosamine-N-Acetyl transferase | 3.2 nmol/mg/h | norm=2.4; aff=0.11 |  | Normal |
| **Enzyme Unit, Chemical Pathology Camelia Botnar laboratories at Great Ormond Street Hospital** | | | | | |
| plasma | Asp-N-acetylglucosaminidase | 161 nmol/24h/ml | 161 | 80-243 | Normal |
| plasma | total β-hexosaminidase | 2.4 µmol/hr/ml | 2.4 | 0.41-1.7 | Raised |
| WBC | α-iduronidase | 51nmol/hr/mg prtn | 51 | 23-92 | Normal |
| WBC | β-Galactosidase | 332 nmol/hr/mg | 332 | 163-378 | Normal |
| plasma | α-N-Acetylglucosaminidase | 72 nmol/hr/ml | 72 | 12-73 | Normal |
| WBC | α-Glucosamine-N-Acetyl transferase | 0.39 nmol/hr/mg | 0.39 | 0.64-4.2 | Decreased |
| WBC | β-Hexosaminidase | 1.68 umol/hr/mg |  |  | Raised |
| WBC | β-Galactosidase (homogenate) | 273 nmol/hr/mg | 273 | 131-303 | Normal |
| Plasma | Iduronate sulphatase | 261 nmol/hr/ml | 261 | 82-324 | Normal |
| plasma | α-fucosidase | 768 nmol/hr/ml | 768 | 175-1403 | Normal |
| plasma | β-Glucuronidase | 93 nmol/hr/ml | 93 | 27-512 | Normal |
| plasma | β-mannosidase | 307 nmol/hr/ml | 307 | 65-677 | Normal |
| plasma | Hexosaminidase A | 305 nmol/hr/ml | 305 | 76-269 | Raised |
| plasma | total β-hexosaminidase | 2.4 |  |  | Raised |
| plasma | α-Mannosidase | 78 nmol/hr/ml | 78 | 19-119 | Normal |
| plasma | Chitotriosidase | 29 nmol/hr/ml | 29 | 0-150 | Normal |
| plasma | α-N-acetylgalactosaminidase | 16.5 | 16.5 | 4.7-26 | Normal |
|  |  |  |  |  |  |

**Figure S1. Absolute neutrophil counts, haemoglobin concentration and total platelets counts in patients homozygous for the *VPS33A* p.R498W mutant.** The middle line in each scatter plots represents mean value. The T-bars represents the standard error of the mean (SEM). The red dotted lines depict normal range in healthy individuals (absolute neutrophil count 1.5-8.5x10^9^/L, platelets count 150-400x10^9^/L). In patients P4 and P5 absolute neutrophil count median 1.3 and 1.5x10^9^/L; range 0.2-3.2x10^9^/ and 0.3-3.9x10^9^/L. Platelet counts median 102 and 70.5 x10^9^/L respectively.

**
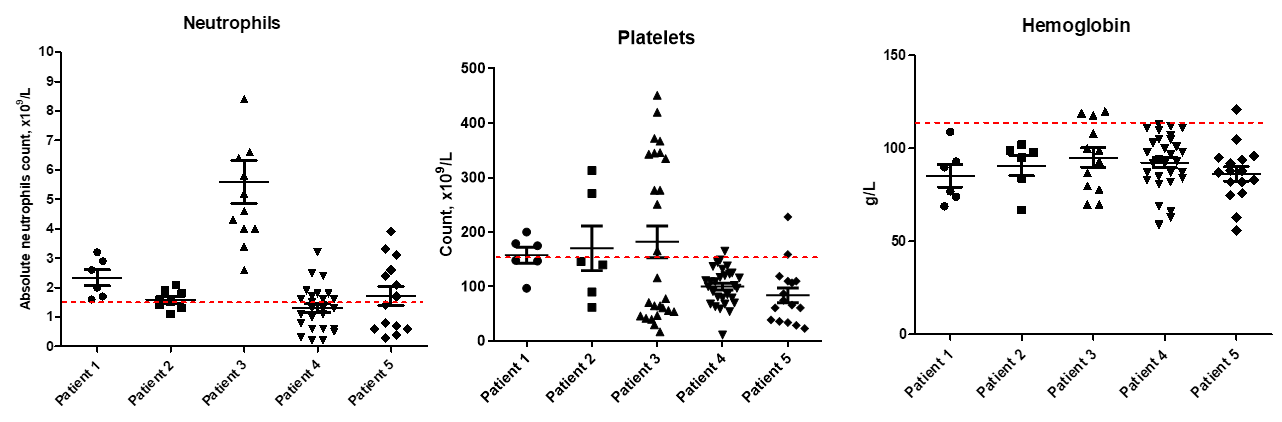
**

**Figure S2.** A) Whole exome sequencing result presented from *Golden Helix GenomeBrowse® visualization tool v2.1.2 by Golden Helix* (1). B) Sanger sequencing of the *VPS33A*. The nucleotide position of cDNA at 1492 is highlighted in blue. The patient 1 is homozygous for c.1492 C>T mutation in *VPS33A* gene. The both healthy parents identified to be heterozygous for the mutation.

VPS33A:NM_022916:c.1492C>T:p.R498W:(13Exons):exon12:missense

**A**

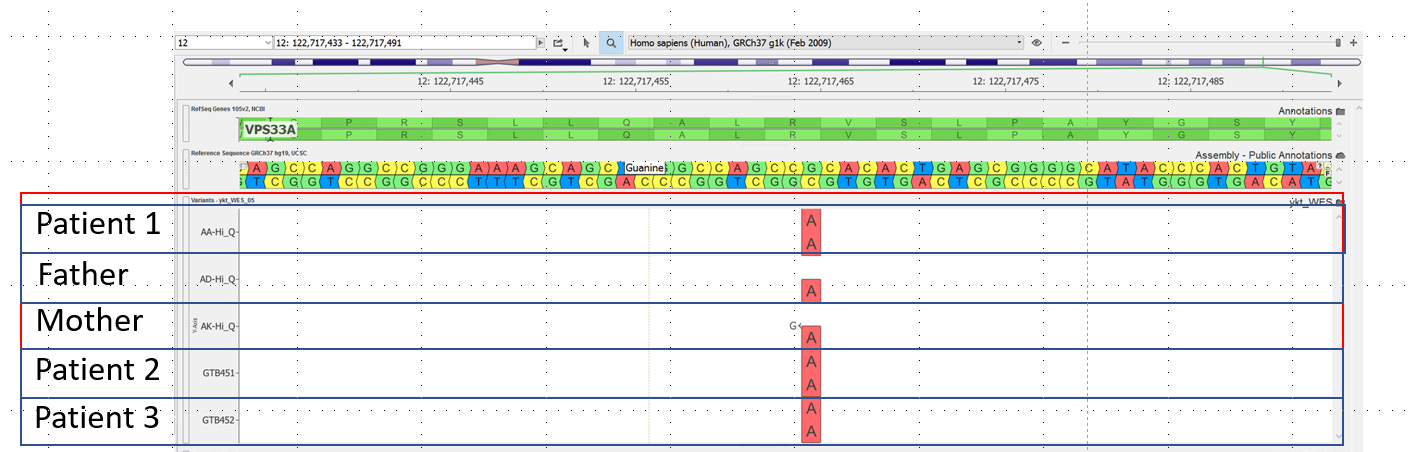


**B**


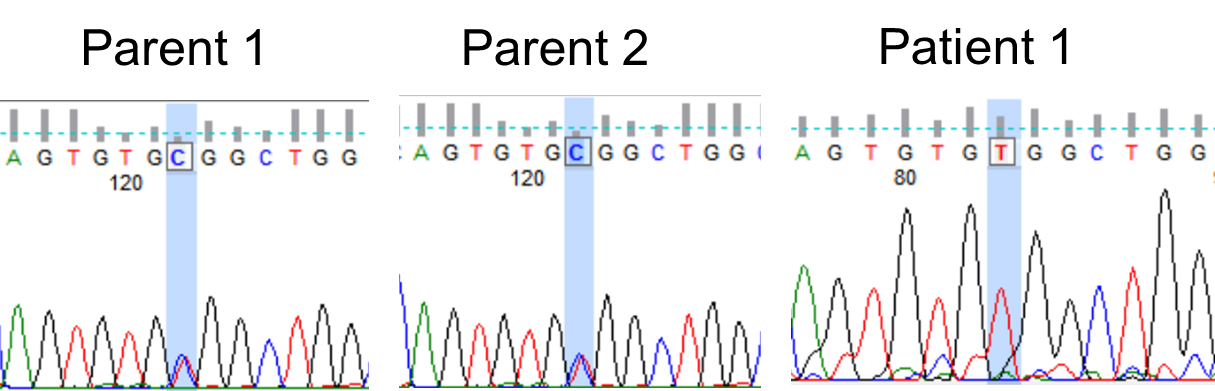


**Table ST2.** Computational relationship analysis in genome-wide study of the single family.

| ID1 | ID2 | Number of SNPs | Z0 | Phi | HetHet IBS0 | Kinship |
| --- | --- | --- | --- | --- | --- | --- |
| Proband P1 | Father | 60799 | 0 | 0.25 | 0.241 0.0003 | 0.2670 |
| Proband P1 | Mother | 60464 | 0 | 0.25 | 0.232 0.0003 | 0.2604 |
| Father | Mother | 60334 | 1 | 0 | 0.171 0.0748 | 0.0242 |

| FID: Family ID for the pair |  |  |  |  | |  | |  | |  | |  |
| --- | --- | --- | --- | --- | --- | --- | --- | --- | --- | --- | --- | --- |
| ID1: Individual ID for the first individual of the pair | |  |  |  | |  | |  | |  | |  |
| ID2: Individual ID for the second individual of the pair | | |  |  | |  | |  | |  | |  |
| N_SNP: The number of SNPs that do not have missing genotypes in either of the individual | | | | | | | | |  | |  | |
| Z0: Pr(IBD=0) as specified by the provided pedigree data | | |  |  | |  | |  | |  | |  |
| Phi: Kinship coefficient as specified by the provided pedigree data | | | | |  | |  | |  | |  | |
| HetHet: Proportion of SNPs with double heterozygotes (e.g., AG and AG) | | | | |  | |  | |  | |  | |
| IBS0: Proportion of SNPs with zero IBS (identical-by-state) (e.g., AA and GG) | | | | |  | |  | |  | |  | |
| Kinship: Estimated kinship coefficient from the SNP data  **Table ST3.** Polymorphisms found in patients homozygous for the *VPS33A* p.R498W mutant.   \|  \|  \|  \|  \|  \|  \|  \|  \| \| --- \| --- \| --- \| --- \| --- \| --- \| --- \| --- \| \| **Chromosome** \| **Position** \| **Gene** \|  \| **ds SNP rs number** \| **Site nucleotide** \| Allele frequency \| **Patients** \| \| 14 \| exon 15 \| *GALC* \|  \| rs398607 \| c.1685T/C (p.I562T) \| 0.44 \| P1, P3 \| \| 14 \| exon 15 \| *GALC* \|  \| rs421466 \| c.1698A/T (p.V566V) \| 0.17 \| P1, P2, P3 \| \| 3 \| signalling peptide \| *GLB1* \|  \| rs7637099 \| c.29C/T (p.L10P) \| 0.55 \| P1 \| \| 3 \| signalling peptide \| *GLB1* \|  \| rs7614776 \| c.44C/T (p.L12L) \| 0.9 \| P1, P2, P3 \|   **Figure S3. Early endosomal marker EEA-1 in MPS plus patient-derived fibroblasts.** Confocal images of cultured patients (P1, P2) and control fibroblasts stained with early endosomal marker EEA-1 Alexa-488 antibody (purple) and DNA (blue) DAPI. A) Patient P1 fibroblasts; B) patient P2 fibroblasts; C) adult control; D) neonatal control. E) EEA-1 Alexa 488 corrected total cell fluorescence quantified in ≥10 fields each contained ≥3 cells. Data presented as Mean ± SD.  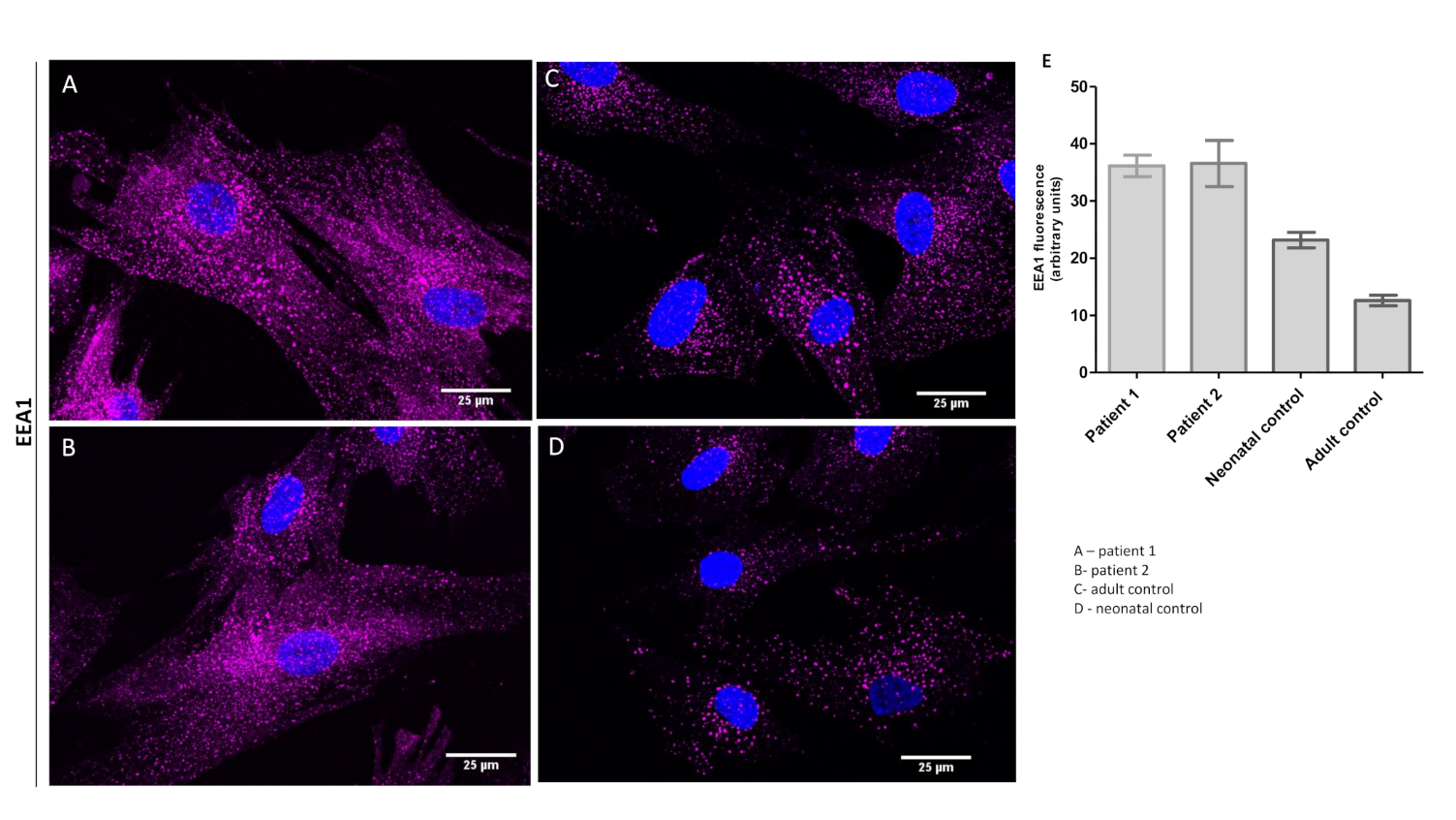    **Figure S4. ~~Staining with~~ Lysosomal markers in MPS plus patient-derived fibroblasts ~~and control cells~~**. A) Representative confocal images of patients and neonatal control fibroblasts stained with anti-LAMP1 and LAMP-2 antibodies (green) and DAPI (blue). B) LAMP-1 and LAMP-2 corrected total fluorescence quantified in ≥ 10 fields as described in methods. Data from three separate experiments presented as Mean ± SD. C) Patient-derived P1, P2 and neonatal control fibroblasts were labelled with acidotropic probes 1µM LysoSensor Blue DND-167 (pK_a_ 5.1) or 100nM LysoTracker Red DND-99. D) LysoSensor DND-167 corrected total cell fluorescence quantified in ≥18 cells per each cell line. Data presented as Mean ± SD, *** - p<0.001.  **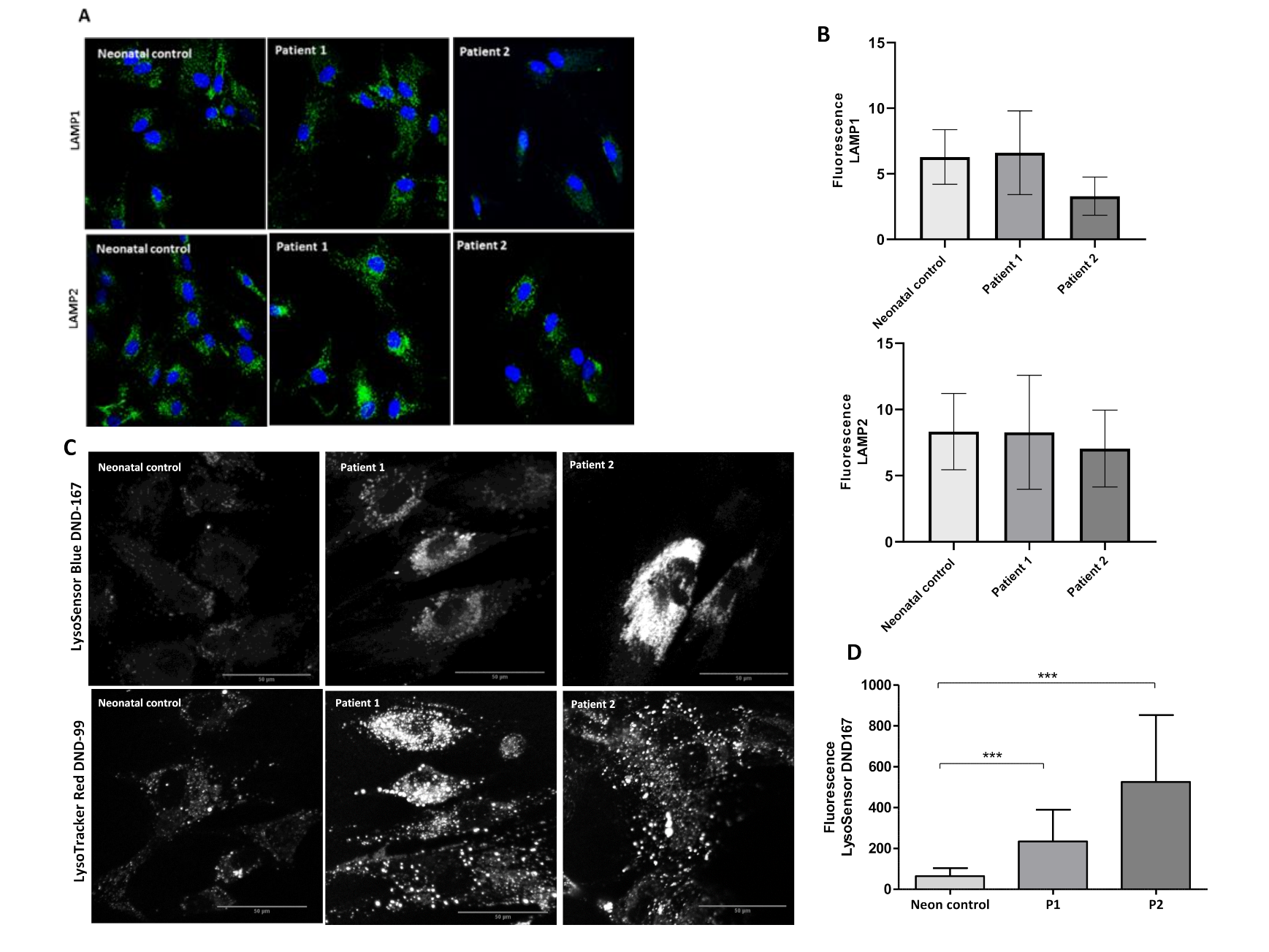**  **Figure S5.** Quantative real-time PCR using TaqMan gene expression assay (Life Technologies). HPRT1 transcripts were used as an endogenous control. 2^-∆∆Ct^ method was used to calculate relative transcript expression. The data presented from three independent experiments shown as Mean ± SD.   | | |  |  | |  | |  | |  | |  |
|  | | | | | | | | | | | | |

**~~Figure S6. Cholesterol accumulation in VPS33A patients.~~** ~~VPS33A patients, Niemann-Pick type C (NPC-1) and control fibroblasts were stained for unesterified cholesterol using filipin. A) Representative confocal images of patients and control cells. B) Quantification of corrected integrated density of fluorescence per area was performed in ≥10 cells. Mean ± SEM *p<0.05.~~

**Figure S6.** Treatment of MPS plus patient-derived fibroblasts (P1, P2) with 50µM miglustat (Zavesca). A) Representative confocal images of BODIPY-LacCer labelled patients (P1 and P2) cells treated with 50µM miglustat for 40 hours. B) Quantification of LacCer puncta in ≥10 cells per each cell line. Data from two independent experiments presented as Mean ± SEM.

**
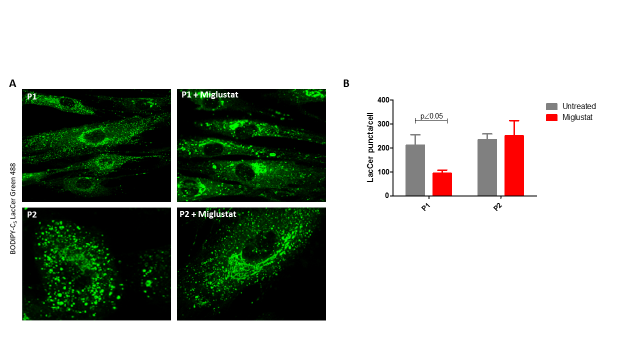
**

**Figure S7. Treatment with 25nM and 100nM eliglustat**. Patient-derived and control fibroblasts were treated with 25nM and 100nM eliglustat tartrate for 24 hours and were labelled with BODIPY-LacCer as described in the methods. A) confocal images of BODIPY-LacCer labelled patients cells treated with 25 and 100nM eliglustat ~~for 24 hours~~. B) and C) Quantification of LacCer puncta in untreated and eliglustat treated cells (n ≥10 cells per each cell type). Data presented as Mean ± SD.

**A**

**
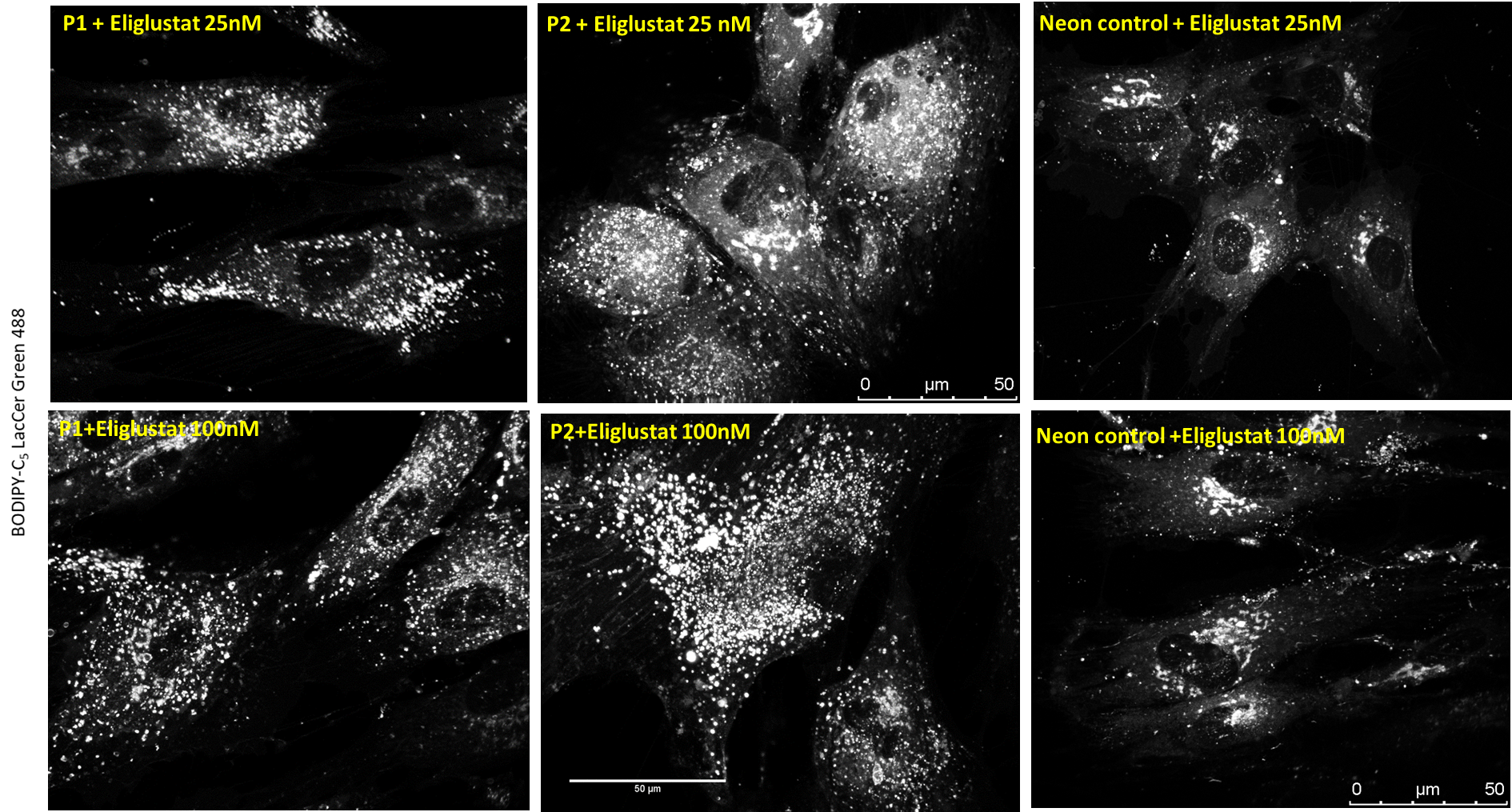
**

**B C**

**References**

1. Golden Helix GenomeBrowse ® visualization tool (Version 2.1.2) [Software]. Bozeman, MT: Golden Helix, Inc. Available from <http://www.goldenhelix.com>
2. GENCODE reference annotation for the human and mouse genomes. Frankish A, Diekhans M, Ferreira AM, Johnson R, Jungreis I, Loveland J, Mudge JM, Sisu C, Wright J, Armstrong J, Barnes I, Berry A, Bignell A, Carbonell Sala S, Chrast J, Cunningham F, Di Domenico T, Donaldson S, Fiddes IT, García Girón C, Gonzalez JM, Grego T, Hardy M, Hourlier T, Hunt T, Izuogu OG, Lagarde J, Martin FJ, Martínez L, Mohanan S, Muir P, Navarro FCP, Parker A, Pei B, Pozo F, Ruffier M, Schmitt BM, Stapleton E, Suner MM, Sycheva I, Uszczynska-Ratajczak B, Xu J, Yates A, Zerbino D, Zhang Y, Aken B, Choudhary JS, Gerstein M, Guigó R, Hubbard TJP, Kellis M, Paten B, Reymond A, Tress ML, Flicek P. Nucleic Acids Res 2018 : Oct24, PUBMED: 30357393; DOI: 10.1093/nar/gky955

**Programs, Databases and Bioinformatic resources used in the manuscript:**

samtools http://www.htslib.org

vt https://genome.sph.umich.edu/wiki/Vt

kggseq1.0 http://grass.cgs.hku.hk/limx/kggseq/index.php

plink1.9 https://www.cog-genomics.org/plink2/

king2.1.2 http://people.virginia.edu/~wc9c/KING/index.html

finchTV1.4 https://softfamous.com/finchtv/

Golden Helix GenomeBrowse® 2.1.2 http://www.goldenhelix.com.

Genome Browser : http://genome-euro.ucsc.edu

ExAC http://exac.broadinstitute.org/terms

ESP http://evs.gs.washington.edu

DiscovEHR http://www.discovehrshare.com

gnomAD http://gnomad.broadinstitute.org/terms

dbSNP https://www.ncbi.nlm.nih.gov/snp

DDD : https://www.ddduk.org

GENCODE : <https://www.gencodegenes.org>

**Supplementary figures and tables**

**Table ST1.** Lysosomal enzymes activities in patient P1.

**Table ST2.** Glycosphingolipids concentrations in patients and controls fibroblasts measured by Mass Spectrometry.

**Table ST3.** Sphingolipids concentrations in patients with mutated VPS33A and control fibroblasts.

**Table ST4.** Computational relationship analysis in genome-wide study of the single family.

**Table ST5.** Polymorphisms found in patients with homozygous mutation c.1492C>T (p.R498W) in the *VPS33A* gene.

**Figure S1.** Distributions of absolute neutrophil counts, haemoglobin concentration and total platelets counts in patients with the homozygous mutation p.R498W in the VPS33A gene.

**Figure S2.** (A) Whole exome sequencing result presented from *Golden Helix GenomeBrowse® visualization tool v2.1.2 by Golden Helix* (1) (B)Sanger sequencing of the VPS33A. The nucleotide position of cDNA at 1492 is highlighted in blue. Patient 1 is homozygous for c.1492 C>T mutation in VPS33A gene. Both healthy parents were identified as heterozygous for the mutation.

**Figure S3. Early endosomal marker EEA-1 distribution.** Representative confocal images of cultured patients and control fibroblasts stained with Alexa-488 (purple) early endosomal marker EEA-1 and DNA (blue) DAPI antibodies: (A) Patient 1 fibroblasts; (B) patient 2 fibroblasts; (C) adult control; (D) neonatal control. E – Corrected integrated density of EEA1-1 Alexa 488 fluorescence per cell area was measured in ≥20 cells per each cell line. The data presented as Mean ± SEM. *p=0.01; **p<0.0001

**Figure S4. Staining with lysosomal markers in patients and control cells**. A) Representative confocal images of patient and neonatal control fibroblasts stained with anti-LAMP1 and LAMP-2 antibodies. B) LAMP-1 and LAMP-2 corrected integrated fluorescence per area quantified in ≥ 7 fields (each contains 3-5 cells) from three independent experiments. Data presented as Mean ± SEM. C) Representative confocal images of patient 1 fibroblasts and neonatal control shown increased acidotropic probe LysoSensor blue DND-167 (pK_a_ 5.1) fluorescence intensity in patient 1 cells.

**Figure S5. Quantitative real-time PCR using TaqMan gene expression assay (**Life Technologies). HPRT1 transcripts were used as an endogenous control. 2^-∆∆Ct^ method was used to calculate relative transcript expression. The data presented from three independent experiments shown as Mean ± SD.

**Figure S6. Treatment of patients cells with 50µM miglustat (Zavesca).** A) Representative confocal images of BODIPY-LacCer labelled patients cells treated with 50µM Miglustat . B) Quantification of LacCer puncta in untreated and Miglustat treated patient cells. Mean ± SEM.

**Figure S7.** **Treatment with 25nM and 100nM eliglustat**. A) confocal images of BODIPY-LacCer labelled patients cells treated with 25 and 100nM eliglustat for 24 hours. B) Quantification of lacer puncta in untreated and eliglustat treated patients and control cells. Data presented as Mean ± SD
